# Enhanced Oral Bioavailability and Pharmacokinetics of Altrenogest via a Self-Nanoemulsifying Drug Delivery System

**DOI:** 10.3390/pharmaceutics18101336

**Published:** 2026-10-21

**Authors:** Yixing Chen, Jing Li, Nanxin Li, Awn Abbas, Xiaoyang Ai, Zhirong Zhang, Wei Zhang, Hualin Fu

**Affiliations:** Department of Pharmacy, College of Veterinary Medicine, Sichuan Agricultural University, Chengdu 611130, China; cheneason83@gmail.com (Y.C.); 15108387481@163.com (J.L.); linanxinao@163.com (N.L.); awni1115@gmail.com (A.A.); a1600063002@163.com (X.A.); 18848219218@163.com (Z.Z.); zhangwei26510c@126.com (W.Z.)

**Keywords:** altrenogest, self-nanoemulsifying system, in vivo absorption, cellular uptake

## Abstract

**Background**: Altrenogest (ALT) is a synthetic progestogen used to synchronize the oestrus of swine and horses. It is poorly absorbed orally and this severely limits its use. Self-nanoemulsifying drug delivery systems (SNEDDSs) have been identified as a potentially efficacious solution to these challenges, as they spontaneously generate fine oil-in-water nanoemulsions upon exposure to gastrointestinal fluids, thereby augmenting the dissolution and bioavailability of lipophilic drugs. **Objective**: In order to enhance the oral bioavailability of ALT, this study developed an ALT-loaded SNEDDS. The mechanisms of cellular uptake were investigated using MDCK monolayers, while in vivo pharmacokinetics and in situ intestinal perfusion studies in rats were conducted to assess systemic absorption, lymphatic transport potential, and intestinal permeation. **Methods & Results**: In vitro, ALT-SNEDDS demonstrated no significant toxicity towards MDCK cells at concentrations up to 12.8 μg/mL. The uptake of ALT-SNEDDS was found to be time- and energy-dependent, involving clathrin- and caveolae-mediated endocytosis. In rats, ALT-SNEDDS and its hydrated form, in comparison with an oil-based formulation, resulted in a significant elevation of C_max_ and AUC, yielding 1.96- and 4.97-fold improvements in oral bioavailability. Lymphatic involvement was supported by a significant decrease in plasma concentration following cycloheximide blockade. Intestinal perfusion showed significantly higher Ka and P_eff_ values for ALT-SNEDDS than free ALT, confirming enhanced small intestinal absorption. In an acute oral toxicity study, a single 40 mg/kg oral dose caused no apparent histopathological abnormalities in the major organs of rats. **Conclusion**: Collectively, the SNEDDS platform markedly facilitates ALT absorption and exhibits lymphatic transport propensity, providing a foundation for subsequent formulation optimization and for evaluation in the target species.

## 1. Introduction

Altrenogest (ALT) is a synthetic oral steroidal progestin widely used in veterinary reproductive management, particularly for estrus synchronization in pigs and horses [1,2]. Its pharmacological effect is primarily mediated through progesterone receptor-dependent negative feedback on the hypothalamic–pituitary–gonadal axis. By suppressing gonadotropin, releasing hormone (GnRH) secretion and subsequently reducing follicle-stimulating hormone (FSH) and luteinizing hormone (LH) release, ALT delays follicular development and prevents estrus during treatment [3,4]. After drug withdrawal, pituitary activity resumes, circulating gonadotropins increase, and synchronized follicular maturation leads to more predictable estrus and ovulation [5]. This endocrine control makes ALT valuable for improving breeding management, reproductive scheduling, and herd level reproductive efficiency.

Despite its practical importance, the oral performance of ALT is limited by its unfavorable physicochemical properties. ALT is highly lipophilic and poorly soluble in water, with an aqueous solubility of approximately 0.04 mg/mL at 25 °C and a logP of 3.9, classifying it as a typical Biopharmaceutics Classification System class II drug [6,7]. Poor aqueous solubility can restrict dissolution in gastrointestinal fluids, resulting in incomplete or variable absorption after oral administration. Conventional dosage forms, including tablets, soft capsules, and oil solutions, are used clinically but do not fully address the dissolution and absorption barriers associated with lipophilic steroidal drugs.

Several formulation strategies have been explored to improve ALT delivery. Injectable phospholipid-based gels have high drug loading capacity and sustained in vivo release, while cyclodextrin inclusion complexes have markedly improved ALT solubility in vitro [8,9]. Ethyl cellulose-based microcapsules have also demonstrated favorable release behavior [10]. Although these approaches provide useful formulation concepts, many involve relatively complex preparation procedures, higher excipient costs, or parenteral delivery routes. More importantly, most available studies focus on formulation characterization and in vitro release, whereas detailed evidence explaining how oral ALT formulations improve intestinal absorption, epithelial uptake, and lymphatic transport remains limited.

Self-microemulsifying drug delivery systems (SMEDDSs) offer a promising strategy for enhancing the oral delivery of poorly water-soluble and lipophilic compounds [11]. SMEDDSs are isotropic mixtures of drug, oil, surfactant, and cosurfactant that spontaneously form fine oil-in-water microemulsions after dilution in gastrointestinal fluids under gentle agitation [12]. The nanoscale droplets generated during self-emulsification provide a large interfacial surface area, maintain the drug in a solubilized state, and improve its contact with the intestinal mucosa. Beyond solubilization, lipid-based systems may enhance absorption by modifying epithelial membrane permeability, reducing efflux transporter activity, and promoting association with lipid digestion products. For sufficiently lipophilic drugs, these systems may also facilitate chylomicron-mediated intestinal lymphatic transport, thereby partially bypassing hepatic first-pass metabolism and improving systemic exposure [13].

Self-emulsifying drug delivery systems (SEDDSs) can be further subclassified into self-microemulsifying drug delivery systems (SMEDDSs) and self-nanoemulsifying drug delivery systems (SNEDDSs) based on the droplet size of the dispersion formed upon aqueous dilution [14]. Although the precise size boundaries vary across different reports, it is generally recognized that SMEDDSs typically form microemulsions with droplet sizes in the range of 100–250 nm, whereas SNEDDSs form nanoemulsions with droplet sizes below 100 nm [15]. Some of the literature further defines SNEDDSs as having droplet sizes between 10 and 200 nm [16]. In recent years, a growing number of researchers have preferred the term “SNEDDS” to emphasize the nanoscale nature of the formulation and its advantages in enhancing the oral absorption of poorly soluble drugs.

These characteristics make SNEDDSs particularly suitable for ALT because of its steroidal structure, high lipophilicity, and dissolution-limited oral absorption. However, increased plasma exposure alone is insufficient to define the absorption mechanism. A more rigorous evaluation requires integration of cellular uptake studies, pharmacokinetic analysis, lymphatic transport assessment, and direct intestinal permeability testing.

This study aimed to develop an ALT-loaded self-nanoemulsifying drug delivery system and systematically investigate its oral absorption behavior. MDCK cell monolayers were used to evaluate cytotoxicity, cellular uptake, and potential uptake pathways. Rat pharmacokinetic experiments were performed to compare systemic exposure after ALT-SNEDDS and conventional oil-based administration, while cycloheximide-mediated lymphatic blockade was used to assess the contribution of intestinal lymphatic transport. In situ intestinal circulation perfusion was further conducted to determine intestinal absorption rate and effective permeability. This integrated approach was designed to clarify whether SNEDDS enhances ALT oral bioavailability through improved solubilization, epithelial uptake, intestinal permeability, and lymphatic transport, providing a mechanistic basis for further formulation optimization and veterinary application.

## 2. Materials and Methods

### 2.1. Materials

Altrenogest reference standard (98%), Krebs-Ringer powder (K-R powder), and Coumarin 6 (Cou6) were obtained from (Shanghai Yuanye Bio-Technology Co., Ltd., Shanghai, China) Altrenogest (ALT) active pharmaceutical ingredient was obtained from (Ningbo Sansheng Biological Technology Co., Ltd., Ningbo, China) Acetonitrile (chromatographic grade) was purchased from (Tianjin Kemiou Chemical Reagent Co., Ltd., Tianjin, China) Cycloheximide (CHX) were purchased from (Shanghai Aladdin Biochemical Technology Co., Ltd., Shanghai, China) Diethylene glycol monoethyl ether (Transcutol HP) was supplied by Gattefosse, Saint-Priest, France, and kindly provided by (Guangzhou Tianrun Pharmaceutical Co., Ltd., Guangzhou, China) Tween 80, ethyl oleate (EO), phenol red, methyl-β-cyclodextrin, quercetin, chlorpromazine hydrochloride, and colchicine were purchased from (Shanghai Macklin Biochemical Co., Ltd., Shanghai, China) PBS buffer, high-glucose DMEM medium (Thermo Fisher Scientific, Waltham, MA, USA), penicillin/streptomycin for cell culture (100×) (Shanghai Yuanpei Biotechnology Co., Ltd., Shanghai, China), fetal bovine serum (FBS; NEWZERUM Ltd., Christchurch, New Zealand), trypsin-EDTA cell digestion solution, CCK-8 kit (Beyotime Biotechnology, Shanghai, China), and cell-culture-grade DMSO (Beijing Solarbio Science & Technology Co., Ltd., Beijing, China) were also used.

### 2.2. Preparation and Characterization of ALT-SNEDDS

The formulation was obtained through stepwise screening of oil phases, surfactants and co-surfactants based on saturated solubility and excipient compatibility, followed by pseudo-ternary phase diagram construction and central composite design–response surface methodology (CCD-RSM) optimisation; the complete data are given in the Supplementary Materials (Section S1) and have been reported previously [17]. Pseudo-ternary phase diagrams identified EO as the optimal oil phase within a K_m_ range of 1.0–2.5, and CCD-RSM optimisation, using the oil fraction (X_1_) and Km (X_2_) as independent variables and the self-emulsifying time and droplet size as responses, determined the final composition of EO 22.0 wt%, Tween 80 54.2 wt% and Transcutol HP 23.8 wt%.

According to the above formulation proportions, EO, Tween 80, and Transcutol HP were weighed into a centrifuge tube and mixed by vortexing to obtain blank SNEDDS. ALT was then added at a ratio of 20 mg/g to the SNEDDS, followed by vortexing for 1 min, ultrasonication for 3 min, and equilibration in a 37 °C water bath with shaking until ALT was completely dissolved, yielding ALT-SNEDDS [17]. Accurately weighed 1 g of ALT-SNEDDS was added to 100 mL of ultrapure water (dilution factor of 100-fold) and allowed to self-emulsify under magnetic stirring at 100 rpm. The solution became clear and transparent or exhibited a bluish opalescence, indicating the formation of ALT-SNEDDS nanoemulsion (i.e., ALT-SNEDDS-Water), which was used for subsequent particle size, Zeta potential, and morphological characterization [17].

For characterisation, the self-emulsifying time was recorded after 0.1 g of ALT-SNEDDS was added to 10 mL of ultrapure water at 37 °C under magnetic stirring (50 rpm), and the nanoemulsion type was judged by the dye-diffusion method using methylene blue and Sudan III. Droplet size and polydispersity index (PDI) were determined by dynamic light scattering (DLS) and zeta potential by electrophoretic light scattering (ELS) on a Malvern Zetasizer Nano ZS after 100-fold dilution with ultrapure water. Droplet morphology was observed with a JEOL JEM-1400FLASH transmission electron microscope (80 kV) after negative staining with 2% phosphotungstic acid. Dilution stability, temperature stability, drug loading, encapsulation efficiency and in vitro release were evaluated as detailed in the Supplementary Materials (Section S3).

### 2.3. Establishment of the MDCK Cell Monolayer Model

For the cellular uptake model, an MDCK cell suspension was seeded into 12-well plates at 5 × 10^4^ cells/well. The culture medium was replaced every other day after seeding, and the cells were used for cellular uptake experiments after 4 days of culture. For qualitative analysis by confocal laser scanning microscopy, an 18 mm cell coverslip was placed in each well of the 12-well plate before cell seeding, and the cells were then cultured in the plates.

### 2.4. Cytotoxicity Assay

MDCK cells were seeded into sterile 96-well plates at 1 × 10^4^ cells/well, with 100 μL per well, and incubated for 24 h at 37 °C in a 5% CO_2_ incubator. After the cells adhered, the culture medium was removed. ALT-SNEDDS, blank SNEDDS, and raw ALT test solutions were added at concentration gradients calculated as ALT concentrations of 204.8, 102.4, 51.2, 25.6, 12.8, 6.4, 3.2, and 1.6 μg/mL, with 100 μL added to each well. The test solutions were prepared by dilution with complete cell culture medium. Five replicate wells were set for each sample, together with a control group without drug and a blank group without drug or cells. The 96-well plates were incubated for 24 h at 37 °C and 5% CO_2_. After incubation, 20 μL of CCK-8 reagent was added to each well, and the plates were incubated in the dark for 1 h at 37 °C and 5% CO_2_. Absorbance at 450 nm was measured using a microplate reader, and cell viability was calculated as follows:

(1) Cell viability (%) = (absorbance of experimental group − absorbance of blank group)/(absorbance of control group − absorbance of blank group) × 100%

### 2.5. Effect of Different Incubation Times on Cellular Uptake

After MDCK cells seeded in 12-well plates had been cultured for 4 days, the cells were incubated with Cou6 (0.1 μg/mL) or Cou6-labeled ALT-SNEDDS solution (ALT concentration, 2 μg/mL) for 0.5, 1.0, and 2.0 h at 37 °C in a 5% CO_2_ incubator. Cells were also incubated with precooled Cou6-labeled ALT-SNEDDS solution at 4 °C for 1 h. The cells were then washed twice with PBS, digested with 0.25% trypsin-EDTA, and collected into 1.5 mL EP tubes. After centrifugation at 8000 rpm for 10 min, the supernatant was discarded. The cell pellet was resuspended in fresh PBS. The selected excitation/emission wavelengths were E_x_/E_m_ = 488/502 nm, and the cell suspension was analyzed by flow cytometry.

### 2.6. Effect of Endocytosis Inhibitors on Cellular Uptake

After MDCK cells seeded in 12-well plates had been cultured for 4 days, the cells were incubated with quercetin, methyl-beta-cyclodextrin, chlorpromazine, or colchicine at 37 °C for 1 h. Cou6-labeled ALT-SNEDDS solution at the same concentration described above was then added, and the cells were incubated for another 1 h at 37 °C. After incubation, the cells were washed twice with PBS, digested with 0.25% trypsin-EDTA, and collected into 1.5 mL EP tubes. After centrifugation at 8000 rpm for 10 min, the supernatant was discarded, and the cell pellet was resuspended in fresh PBS. The cell suspension was analyzed by flow cytometry. The types and doses of inhibitors are shown in Table 1.

### 2.7. Cell Uptake Assay

MDCK cells seeded in confocal dishes were incubated with Cou6-labeled ALT-SNEDDS solution at 37 °C for 1 h. Another group of MDCK cells was pretreated at 4 °C for 1 h and then incubated with precooled Cou6-labeled ALT-SNEDDS solution at the same concentration for 1 h at 4 °C. After incubation, the cells were washed three times with ice-cold PBS and fixed with 4% paraformaldehyde for 30 min. The cells were then washed twice with PBS, stained with 10 μL DAPI per well for 10 min, washed three more times with PBS, and observed by confocal laser scanning microscopy.

### 2.8. Processing of Rat Plasma Samples

A 0.2 mL plasma sample was transferred to an EP tube, and 0.8 mL ethyl acetate was added. The mixture was vortexed for 3 min and centrifuged at 8200× *g* for 10 min at 4 °C. Then, 0.7 mL of the upper clear layer was transferred to another clean centrifuge tube. Another 0.8 mL ethyl acetate was added to the tube containing the remaining plasma residue, and the above extraction procedure was repeated. The extraction was performed three times in total, and the combined extracts were vacuum-dried. After complete evaporation, 0.1 mL acetonitrile was added to the tube, followed by vortexing for 2 min and ultrasonication for 3 min to redissolve the residue. The sample was centrifuged at 11,800× *g* for 10 min, and the upper clear layer was filtered through a 0.22 μm polytetrafluoroethylene syringe filter. A 20 μL aliquot of the filtrate was injected for HPLC analysis.

The HPLC-UV method was fully validated (specificity, linearity, LOD/LOQ, precision, accuracy and recovery); the validation parameters are provided in the Supplementary Materials (Section S4).

### 2.9. Pharmacokinetic Experiment in Rats

Twenty female SD rats were randomly divided into four groups, with five rats in each group. After one week of acclimatization, the rats were fasted for 12 h before the experiment but allowed free access to water, and water remained available during the experiment. ALT-Oil, ALT-SNEDDS, and ALT-SNEDDS-Water were administered orally by gavage at 40 mg/kg, calculated as ALT. In the lymphatic blockade group, rats first received an intraperitoneal injection of Cycloheximide (CHX) in normal saline at 3 mg/kg, followed 1 h later by gavage administration of ALT-SNEDDS-Water at 40 mg/kg, calculated as ALT. ALT-Oil was prepared by dissolving 200 mg ALT in 10 g caprylic/capric triglyceride and was used as the control group for the commercial ALT oral solution. ALT-SNEDDS-Water was prepared by fully hydrating 3 g ALT-SNEDDS in 30 mL water to obtain a 2 mg/mL ALT-SNEDDS aqueous solution. After dosing, 0.5 mL blood samples were collected from the orbital vein at 0.08, 0.25, 0.5, 1, 2, 4, 6, 8, 10, 12, and 24 h. The blood samples were centrifuged in a high-speed refrigerated benchtop centrifuge at 1600× *g* for 10 min at 4 °C, and the upper plasma layer was collected for sample processing.

The body weights of the Sprague-Dawley rats are 200 ± 15 g. All animal experimental procedures were reviewed and approved by the Ethical Scientific Committee of Sichuan Agricultural University (Chengdu, Sichuan, China; Approval No. SAU-20250036).

### 2.10. In Situ Intestinal Perfusion Experiment in Rats

Blank phenol red K-R buffer was prepared by dissolving K-R powder, 1.37 g anhydrous sodium bicarbonate, and 20 mg phenol red in 1000 mL water. Then, 0.2 g SNEDDS was dissolved in 250 mL K-R buffer to prepare an ALT-SNEDDS test solution at a concentration of 16 μg/mL, calculated as ALT. Separately, 4 mg ALT was dissolved in 250 mL K-R buffer containing 1% Tween 80 to prepare a raw ALT test solution at a concentration of 16 μg/mL, calculated as ALT.

Rats fasted for 12 h but allowed free access to water were anesthetized by intraperitoneal injection of 20% urethane solution at 0.6 mL/100 g and then fixed in position. The abdominal cavity was opened through an approximately 3 cm incision along the abdominal midline. Incisions were made 1 cm distal to the pylorus and 5 cm proximal to the cecum. The intestinal segment was first flushed with 37 °C normal saline until no contents remained, and residual liquid was then expelled with air. Silicone tubes were inserted into both ends of the intestinal segment and ligated with thread. The tubes were connected to a constant-flow pump, and the inlet and outlet silicone tubes were placed together in a conical flask. The circuit was first equilibrated with perfusion solution at a flow rate of 2 mL/min for 10 min. Immediately afterward, 1 mL intestinal perfusate was collected from the conical flask as the 0 h sample for drug and phenol red concentrations, and 1 mL phenol red K-R buffer was added to replace the collected volume. Circulating perfusion continued for 2 h at 2 mL/min. Every 15 min, 1 mL test solution was collected from the conical flask into a 1.5 mL centrifuge tube, and 1 mL phenol red K-R buffer was added. At the end of the experiment, the tested intestinal segment was excised, and its length and internal diameter were measured.

As mentioned previously, in Section 2.9.

The HPLC-UV method for ALT and the spectrophotometric method for phenol red were fully validated; the validation parameters are provided in the Supplementary Materials (Section S5).

### 2.11. Acute Oral Toxicity Study in Rats

Acute oral toxicity was assessed in female Sprague-Dawley rats (180 ± 15 g). Rats were randomly assigned to four groups (n = 6): normal saline control, blank SNEDDS control, ALT-Oil solution and ALT-SNEDDS, and each received a single oral dose of 40 mg ALT per kg by gavage after an overnight fast. Clinical signs and body weight were recorded for 7 days; on day 7 the rats were euthanised, and the heart, liver, spleen, lung and kidney were collected for organ weighing and H&E-stained histopathological examination. All procedures were approved by the Ethical Scientific Committee of Sichuan Agricultural University (Approval No. SAU-20250036); full details are given in the Supplementary Materials (Section S6).

## 3. Results

### 3.1. In Vitro Characterization of ALT-SNEDDS

The drug loading of ALT-SNEDDS was 19.44 ± 0.39 mg/g and the encapsulation efficiency was 98.85 ± 0.69% (n = 3; Table S10). The near-complete encapsulation efficiency confirms that ALT was almost entirely dissolved in the pre-concentrate, with negligible loss upon aqueous dilution, whereas the drug loading is governed by the solubility of ALT in the excipient blend and by the intended dose, rather than by any fixed encapsulation capacity. Given the low clinical dose of ALT, this loading is sufficient to deliver the 40 mg/kg dose used in the pharmacokinetic study within a practicable administration volume.

Upon 100-fold dilution with ultrapure water, the formulation self-emulsified within 35.76 ± 1.10 s (n = 3) to form a clear, bluish nanoemulsion of the O/W type (Figures S5 and S6). The diluted samples exhibited a mean droplet size of 18.39 ± 0.03 nm, a PDI of 0.083 ± 0.090 and a zeta potential of −1.12 ± 0.12 mV (n = 3; Figure S7).

TEM images showed uniformly sized, spherical droplets with no visible aggregation (Figure S8). The clear Tyndall effect observed under red laser illumination further supported nanoemulsion formation. Droplet size remained stable after 10-to-1000-fold dilution, with no phase separation, turbidity, or drug precipitation observed within 12 h (Figure S9).

Collectively, ALT-SNEDDS spontaneously forms uniform nanoscale droplets upon aqueous dilution, providing a solid basis for its oral absorption.

### 3.2. Uptake Mechanism of ALT-SNEDDS in MDCK Cells

#### 3.2.1. Cytotoxicity

**Figure 1:**
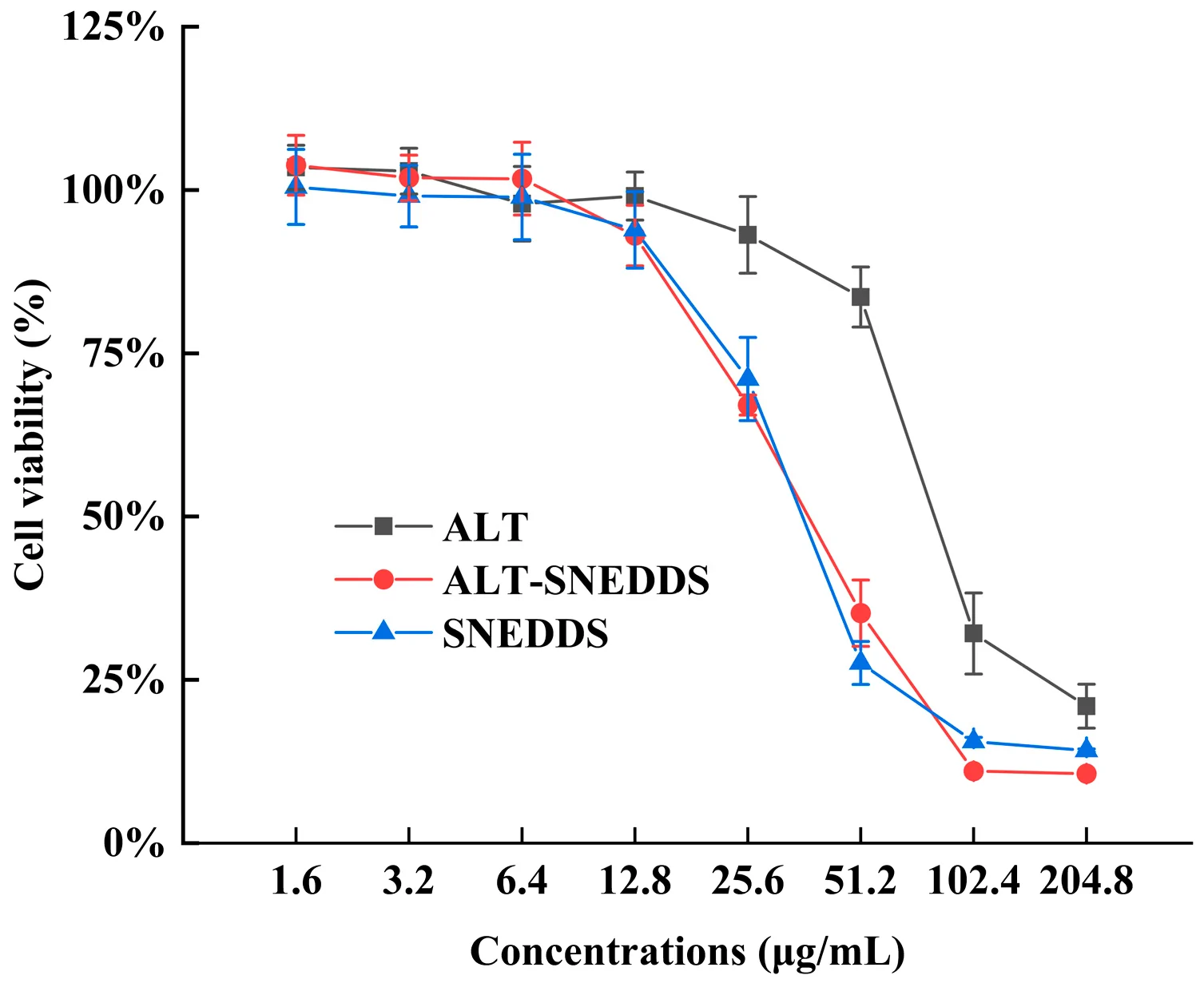
Cytotoxicity of ALT, ALT-SNEDDS, and blank SNEDDS in MDCK cells. MDCK cells were incubated with increasing concentrations (1.6–204.8 μg/mL, based on ALT content) of free ALT, ALT-SNEDDS, or blank SNEDDS for 24 h. Cell viability was determined using the CCK-8 assay and normalized to untreated control cells (set as 100%). Data are presented as mean ± SD (n = 5).

Cell viability exhibited a progressive decline with increasing drug concentration, as it shows in Figure 1. When the administered concentration was 12.8 μg/mL or lower, cell viability remained above 90%; accordingly, subsequent cellular experiments were performed at concentrations not exceeding this threshold. In comparison with both blank SNEDDS and drug-loaded SNEDDS, free ALT showed higher cell viability at equivalent concentrations, implying that the cytotoxicity observed was largely contributed by formulation excipients rather than the drug entity itself. This notion is reinforced by the nearly identical viability profiles of blank and ALT-loaded SNEDDS across the tested concentration spectrum, indicating that incorporation of ALT into the SNEDDS matrix introduces no appreciable cytotoxic burden beyond that attributable to the excipients.

The observed reduction in cell survival at elevated concentrations is most likely driven by Tween 80, the predominant surfactant in the formulation. As a non-ionic surfactant, Tween 80 can insert into the lipid bilayer of cell membranes at higher concentrations, thereby increasing membrane fluidity and permeability. This can ultimately impair membrane integrity and compromise cell viability. This is consistent with previous reports that polysorbate 80 compromises membrane integrity and cell viability in vitro; for example, the extent of membrane rupture induced by polysorbate 80-containing self-emulsifying formulations correlated positively with cytotoxicity in Caco-2 cells [18]. Importantly, while unformulated ALT in suspension may contact the cell membrane directly as solid particles or crystalline aggregates, potentially inducing localized mechanical stress, the entrapment of ALT within the oil-surfactant core of the SNEDDS sequesters the hydrophobic drug inside the nanoemulsion droplets, thereby limiting its direct interaction with the epithelial monolayer. Although such physical encapsulation does not lower the formulation’s overall cytotoxicity, which remains excipient-dominated, it effectively circumvents the additional cell damage that could otherwise arise from exposure to crystalline drug particulates.

#### 3.2.2. Effects of Different Incubation Times and Temperatures on MDCK Cellular Uptake

**Figure 2:**
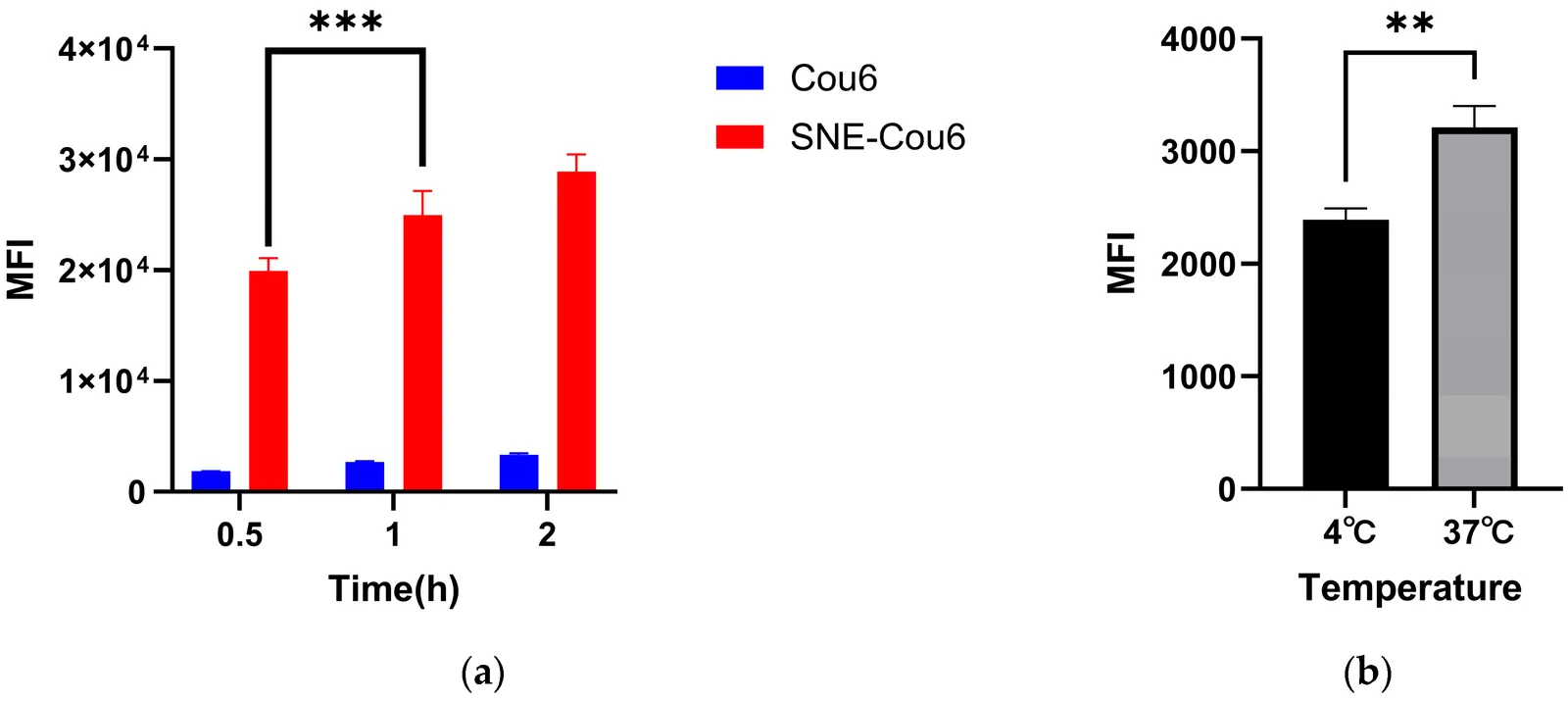
(**a**) Time-dependent uptake of Cou6-labeled ALT-SNEDDS (SNE-Cou6) and free Cou6 in MDCK cells. MDCK cells were incubated with SNE-Cou6 (ALT concentration: 2 μg/mL) or free Cou6 (0.1 μg/mL) at 37 °C for 0.5, 1.0, and 2.0 h. Cellular uptake was quantified by flow cytometry and expressed as mean fluorescence intensity (MFI). Data are presented as mean ± SD (n = 3). Statistical significance was determined using two-way ANOVA followed by Tukey’s post-hoc test. *** *p* < 0.001. (**b**) Temperature-dependent uptake of Cou6-labeled ALT-SNEDDS (SNE-Cou6) in MDCK cells. MDCK cells were incubated with SNE-Cou6 (ALT concentration: 2 μg/mL) at 37 °C or 4 °C for 1 h. Cellular uptake was quantified by flow cytometry and expressed as mean fluorescence intensity (MFI). Data are presented as mean ± SD (n = 3). Statistical significance was determined using two-way ANOVA followed by Tukey’s post-hoc test ** *p* < 0.01.

As shown in Figure 2a, the mean intracellular fluorescence intensity increased with incubation time. The mean fluorescence intensity (MFI) at 0.5 h differed significantly from that at 1 h, whereas there was no significant difference between 2 h and 1 h. This indicates that cellular uptake of SNEDDS increased with time and reached saturation after 1 h. In addition, compared with the free Cou6 group, the MFI of the self-nanoemulsion group was higher at all three time points, 0.5, 1, and 2 h, with significant differences (*p* < 0.001). This suggests that Cou6 did not affect intracellular uptake of SNEDDS and that Cou6-loaded SNEDDS can represent the intracellular uptake behavior of SNEDDS.

Low temperature (4 °C) inhibits ATP-dependent cellular processes. When intracellular ATP is depleted, energy-dependent endocytosis is inhibited. Figure 2b shows that lowering the temperature from 37 °C to 4 °C significantly reduced the mean fluorescence intensity in MDCK cells. This indicates that SNEDDS uptake by cells largely depends on temperature or energy and further suggests that the cellular uptake process may involve active transport.

#### 3.2.3. Effects of Inhibitors of Different Uptake Pathways on MDCK Cellular Uptake

**Figure 3:**
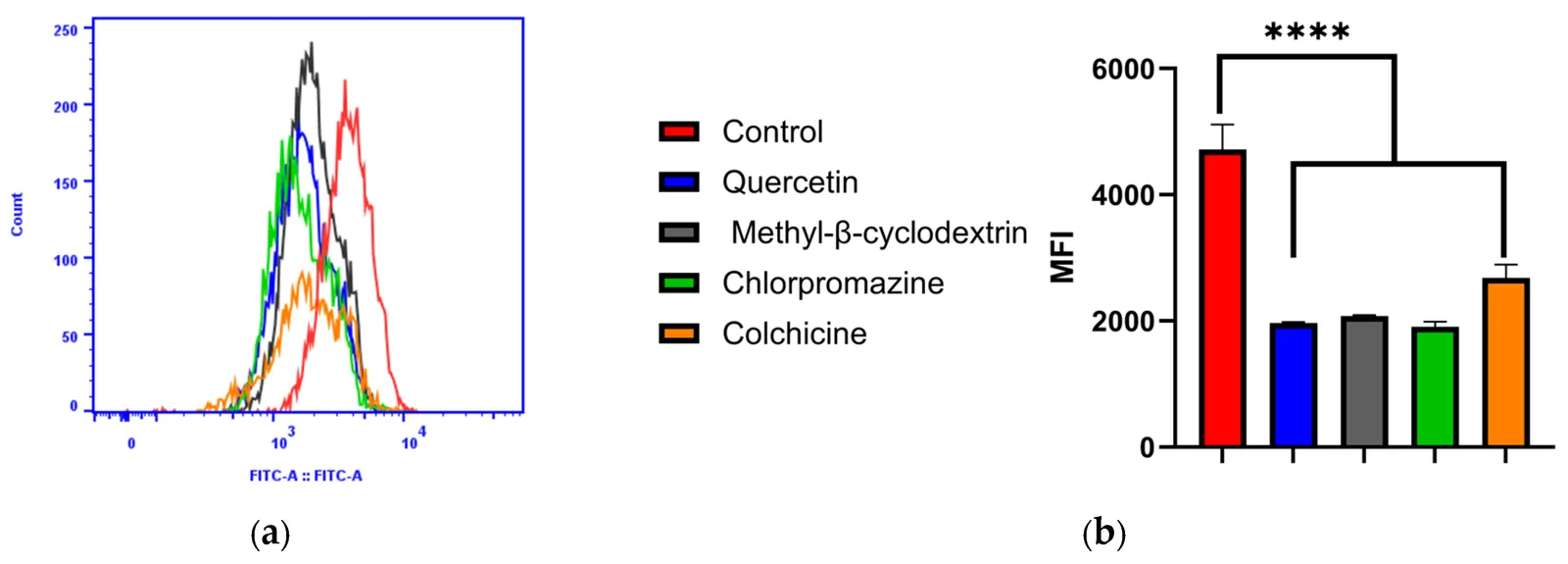
Effects of endocytic pathway inhibitors on the cellular uptake of Cou6-labeled ALT-SNEDDS in MDCK cells. (**a**) MDCK cells were pre-incubated with quercetin (20 μg/mL, lipid raft inhibitor), methyl-β-cyclodextrin (10 mmol/L, caveolae inhibitor), chlorpromazine (30 μmol/L, clathrin inhibitor), or colchicine (1 μmol/L, macropinocytosis inhibitor) for 1 h, followed by incubation with SNE-Cou6 (ALT concentration: 2 μg/mL) for an additional 1 h. Cellular uptake was quantified by flow cytometry and expressed as mean fluorescence intensity (MFI) relative to the untreated control group (set as 100%). (**b**) Data is presented as mean ± SD (n = 3). Statistical significance was determined using two-way ANOVA followed by Tukey’s post-hoc test. **** *p* < 0.0001.

As shown in Figure 3, the intracellular fluorescence intensity in all four endocytosis inhibitor groups was significantly lower than that in the control group (*p* < 0.001), whereas no significant differences were observed among the inhibitor groups. These results indicate that uptake of SNEDDS by MDCK cells may be associated with clathrin-mediated endocytosis, caveolin-mediated endocytosis, lipid raft-mediated endocytosis, and macropinocytosis, suggesting the involvement of all four endocytic pathways.

#### 3.2.4. Qualitative Analysis of Cellular Uptake by Confocal Laser Scanning Microscopy

**Figure 4:**
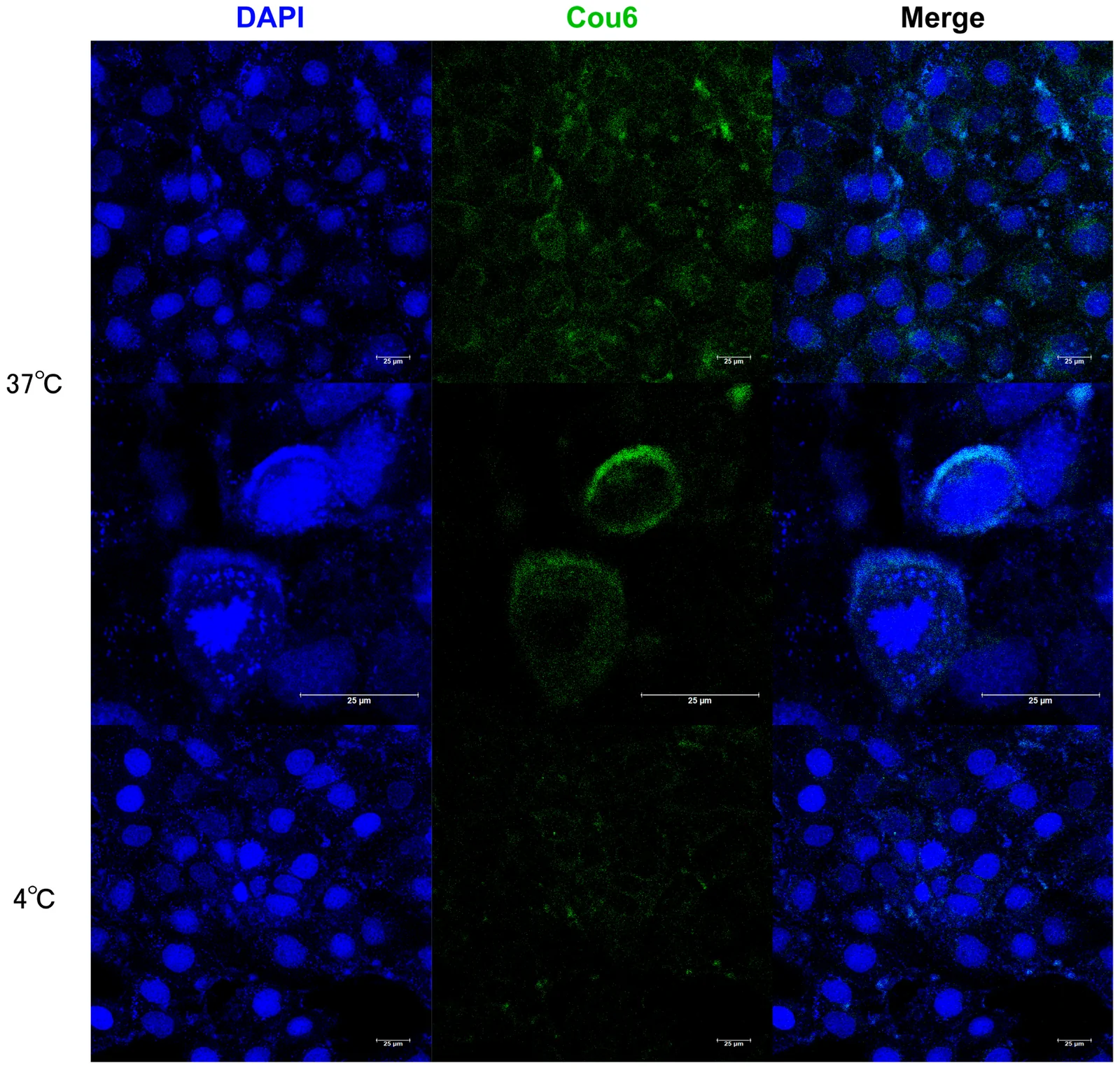
Confocal laser scanning microscopy images of Cou6-labeled ALT-SNEDDS uptake in MDCK cells. MDCK cells were incubated with SNE-Cou6 (ALT concentration: 2 μg/mL) at 37 °C (upper and middle panels) or 4 °C (lower panels) for 1 h. Cell nuclei were stained with DAPI (blue), and Cou6 fluorescence is shown in green. Scale bar = 25 μm.

Confocal laser scanning microscopy was used to examine the cellular uptake of Cou6-labeled ALT-SNEDDS, as shown in Figure 4. DAPI stained the cell nuclei, which appeared as blue fluorescence, whereas Cou6 appeared as green fluorescence. The first row of images shows normal uptake of ALT-SNEDDS at 37 °C. To present cellular uptake more clearly, the field of view was enlarged, and the second row of images was captured. Green fluorescence was concentrated around the nuclei, indicating that many ALT-SNEDDS droplets were taken up by MDCK cells. The third row of images shows that intracellular fluorescence intensity at 4 °C was markedly weaker than that in the normal uptake group at 37 °C, which is consistent with the flow cytometry results.

### 3.3. In Vivo Pharmacokinetics of ALT-SNEDDS in Rats

**Figure 5:**
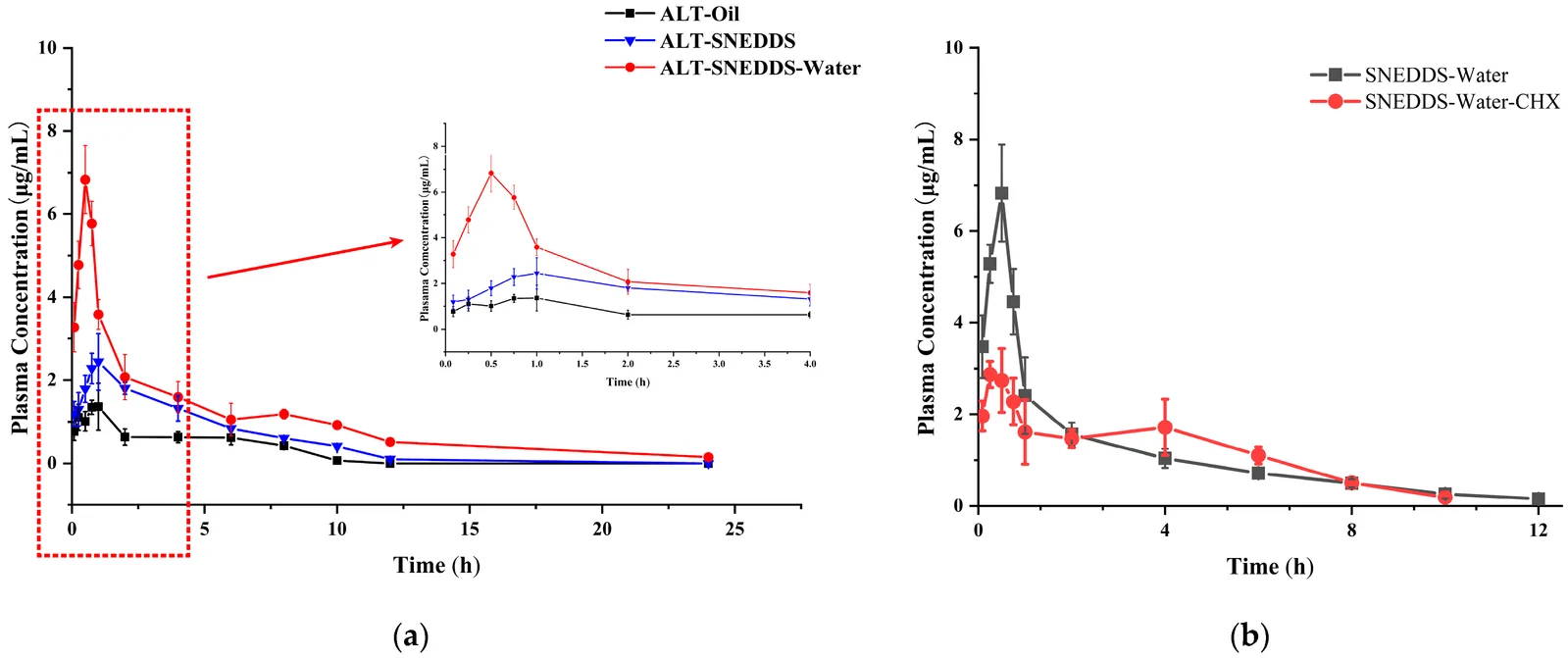
(**a**) Mean plasma concentration–time profiles of ALT in rats following oral administration of ALT-Oil, ALT-SNEDDS, and ALT-SNEDDS-Water. Rats (n = 5) were orally administered ALT-Oil, ALT-SNEDDS, or ALT-SNEDDS-Water at a dose of 40 mg ALT/kg. Blood samples were collected at designated time points (0.08–24 h post-administration), and plasma ALT concentrations were determined by HPLC. Data are presented as mean ± SD. (**b**) Effect of cycloheximide (CHX)-induced lymphatic blockade on plasma concentration–time profiles of ALT-SNEDDS-Water in rats. Rats were orally administered ALT-SNEDDS-Water (40 mg ALT/kg) with or without prior intraperitoneal injection of CHX (3 mg/kg). CHX was administered 1 h before oral dosing. Blood samples were collected at designated time points (0.08–12 h post-administration), and plasma ALT concentrations were determined by HPLC. Data are presented as mean ± SD (n = 5).

Following oral administration of ALT-Oil, ALT-SNEDDS, and ALT-SNEDDS-Water to rats, analysis of the plasma concentration–time data using DAS 2.0 showed that all formulations followed a two-compartment model and underwent rapid intestinal absorption. The plasma ALT concentration–time curves and pharmacokinetic parameters are shown in Figure 5a and Table 2, respectively. Peak concentration (C_max_) is the maximum blood concentration reached after the drug enters the bloodstream following administration. Based on C_max_, the ALT-SNEDDS group (1.33 ± 0.49 μg/mL) and ALT-SNEDDS-Water group (6.83 ± 1.06 μg/mL) both differed significantly from the ALT-Oil group (0.87 ± 0.21 μg/mL), indicating that ALT-SNEDDS significantly increased the plasma ALT concentration in rats compared with the commercial oil formulation. AUC, the area under the drug concentration–time curve, is proportional to the total amount of drug absorbed and reflects the extent of absorption. A larger AUC indicates more complete absorption in the organism. The AUC_(0-t)_ values of the ALT-SNEDDS group (5.14 ± 1.89 μg/mL·h) and ALT-SNEDDS-Water group (13.03 ± 1.42 μg/mL·h) differed significantly from that of the ALT-Oil group (2.62 ± 0.94 μg/mL·h), indicating that ALT-SNEDDS increased the in vivo absorption of ALT and improved bioavailability by 1.96-fold and 4.97-fold, respectively. Notably, C_max_ and AUC in the ALT-SNEDDS-Water group also differed significantly from those in the ALT-SNEDDS group, indicating that hydration and dilution of ALT-SNEDDS enhanced oral ALT absorption. This may be because rats are small and have limited gastrointestinal fluid volume; after entering the gastrointestinal tract, ALT-SNEDDS may not emulsify sufficiently and may require a longer emulsification time, leading to lower plasma concentrations than those achieved after direct dilution before administration.

The AUC of ALT-SNEDDS-Water without pretreatment represents ALT absorbed through all pathways, including the portal vein and lymphatic transport. After intraperitoneal injection of Cycloheximide (CHX), the AUC represents ALT absorbed through pathways other than lymphatic transport. This portion of ALT is absorbed through epithelial cells and directly enters the blood circulation. The plasma concentration–time curve and pharmacokinetic parameters after intraperitoneal injection of 3 mg/kg CHX followed by oral ALT-SNEDDS-Water administration are shown in Figure 5b and Table 3. After intraperitoneal CHX injection, the plasma ALT concentration decreased significantly, and both C_max_ and AUC_(0−2h)_ differed significantly from those in the group not treated with CHX (*p* < 0.05), indicating that ALT-SNEDDS-Water showed a tendency toward lymphatic transport. According to the literature, the in vivo elimination model of intraperitoneally injected CHX is a one-compartment model, with a t_1/2_ of 4.19 h. Because oral administration was performed 1 h after intraperitoneal CHX injection, the plasma concentration–time curve showed a rebound in plasma drug concentration in the lymphatic blockade group at 4 h after dosing. At this point, more than 50% of CHX had been eliminated from the body, and the blocking effect had weakened, causing the plasma concentration to increase.

### 3.4. In Situ Intestinal Absorption Characteristics of ALT-SNEDDS

**Figure 6:**
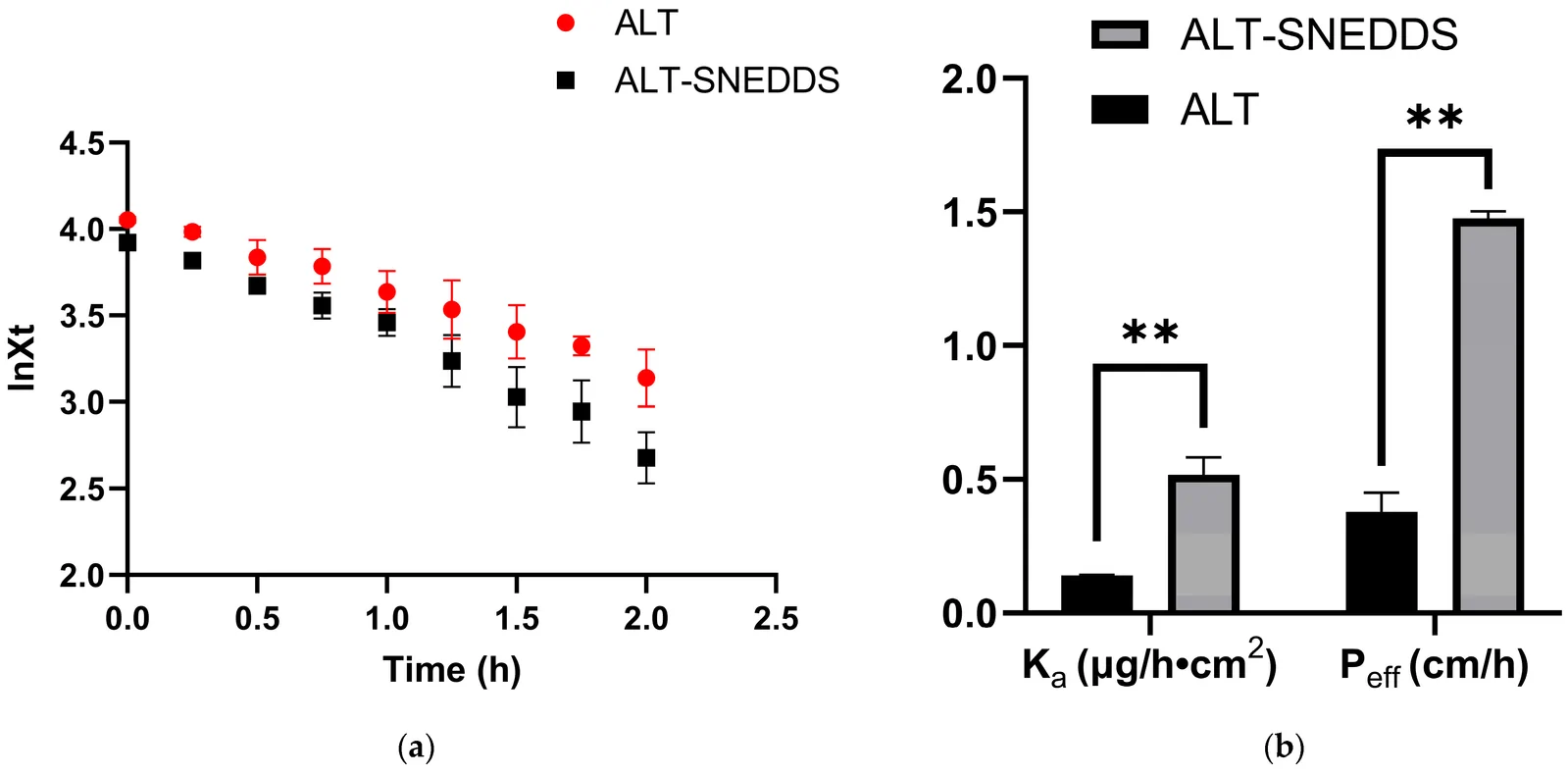
(**a**) Log-linear plot of residual ALT amount versus time in the in-situ rat intestinal perfusion model. Rats were subjected to in situ intestinal perfusion with ALT-SNEDDS or free ALT solution (both at 16 μg/mL ALT in K-R buffer containing phenol red). The logarithm of the remaining drug amount (lnX_t_) in the perfusate was plotted against perfusion time (t). Data are presented as mean ± SD (n = 5). (**b**) Comparison of absorption rate constant (K_a_) and effective permeability (P_eff_) between ALT-SNEDDS and free ALT in the rat in situ intestinal perfusion model. K_a_ and P_eff_ values were calculated from the in situ intestinal perfusion data in rats (n = 5). Data are presented as mean ± SD. Statistical significance was determined using two-way ANOVA followed by Tukey’s post-hoc test. ** *p* < 0.01.

The logarithm of the residual amount of ALT, X_t_ (μg), in the intestinal perfusate was linearly regressed against sampling time, t (h). According to the equation lnX_t_ = x − K_a_t, the slope of the resulting line is K_a_. In situ cyclic perfusion was continued for 2 h, and samples were collected every 15 min. The log residual dose-time curve of ALT during perfusion is shown in Figure 6.

K_a_ represents the rate at which a component enters the systemic circulation from the absorption site and reflects the speed of absorption in the intestinal segment. A larger K_a_ indicates faster absorption in that segment. The absorption rate constant of the ALT-SNEDDS group was 0.52, with lnX_t_ = −0.5211t + 4.0209, whereas that of the Raw ALT group was 0.14, with lnX_t_ = −0.1449t + 3.9483. P_eff_ represents the ability of a component to permeate biological membranes and reflects the strength of absorption in different intestinal segments. In Table 4, both K_a_ and P_eff_ were markedly higher in the ALT-SNEDDS group than in the raw ALT group, indicating that ALT-SNEDDS enhanced drug absorption in the small intestine and thereby improved the oral bioavailability of ALT.

**Table 4:** K_a_ and P_eff_ of ALT-SNEDDS/ALT (n = 5). Data are presented as mean ± SD. Statistical significance was determined using two-way ANOVA followed by Tukey’s post-hoc test. ** *p* < 0.01.

Group	K_a_ (μg/h∙cm^2^)	100 × P_eff_ (cm/h)
ALT-SNEDDS	0.52 ± 0.11 **	149.43 ± 3.76 **
ALT	0.14 ± 0.03	34.23 ± 1.29

### 3.5. Acute Oral Toxicity and Histopathological Evaluation

No mortality or abnormal clinical signs occurred in any group during the 7-day observation, and body weight increased normally in all groups without significant differences (Figure S20). The organ coefficients of the heart, liver, spleen, lung and kidney were comparable among groups (Table S17). Histologically, the myocardium, hepatic lobules, alveoli, splenic red and white pulp, and renal glomeruli and tubules in the ALT-SNEDDS group all retained normal architecture, with no necrosis, inflammatory infiltration, fatty degeneration, fibrosis or haemorrhage relative to the control group (Figure S21). Taken together, a single oral dose of 40 mg/kg, roughly 100-fold higher than the clinical dose, caused no apparent acute toxicity or histopathological injury to the major organs examined.

## 4. Discussion

The ALT self-emulsifying formulation prepared in this study, upon 100-fold dilution with ultrapure water, exhibited a mean particle size of 18.39 ± 0.03 nm, a polydispersity index of 0.083 ± 0.090, and a Zeta potential of −1.12 ± 0.12 mV. TEM revealed spherical and uniformly distributed droplets without observable adhesion [17]. According to the particle size-based classification of self-emulsifying formulations, systems with droplet sizes below 100 nm upon dispersion are defined as self-nanoemulsifying drug delivery systems (SNEDDSs), whereas those with droplet sizes in the range of 100–250 nm are categorized as self-microemulsifying drug delivery systems (SMEDDSs) [15]. Some of the literature further defines SNEDDSs as having droplet sizes between 10 and 200 nm [16]. Although the precise size boundaries vary across reports, the 18.39 nm droplet size of the present formulation is well below the 100 nm threshold, clearly falling within the SNEDDS category. Therefore, to accurately reflect the nanoscale nature of the formulation and to maintain consistency with current terminological conventions, the term “SNEDDS” rather than “SMEDDS” is used uniformly throughout this manuscript. The formation of such nanoscale droplets is attributed to the effective reduction in oil–water interfacial tension by Tween 80 (54.2 wt%) and the synergistic promotion of interfacial film formation by Transcutol HP (23.8 wt%) [17], enabling the system to spontaneously generate thermodynamically stable nanoemulsions with uniform droplet size upon aqueous dilution. The extremely small droplet size provides a vast specific surface area, facilitating the dissolution rate of ALT; meanwhile, the narrow size distribution ensures uniformity of droplet behavior, providing a structural foundation for subsequent investigations of cellular uptake, intestinal absorption, and lymphatic transport.

In the rat pharmacokinetic experiment of ALT-SNEDDS, the drug concentration–time data fitted a two-compartment model and showed rapid transintestinal absorption. This pharmacokinetic behavior is consistent with the expected performance of SNEDDS, which can spontaneously form nanoscale oil-in-water dispersions in gastrointestinal fluids and thereby improve the dissolution and absorption of poorly water-soluble lipophilic drugs. Compared with previously reported ALT formulation strategies, including injectable phospholipid gels [8], cyclodextrin inclusion complexes, and ethyl cellulose microcapsules [4], the present study provides stronger in vivo evidence because it does not only improve formulation performance but also clarifies systemic absorption, intestinal permeability, lymphatic involvement, and cellular uptake mechanisms.

The absorption half-life (T_1/2Ka_) is an important indicator of drug absorption rate. The results showed that T_1/2Ka_ in the ALT-SNEDDS-Water group was lower than that in the ALT-Oil group, indicating that the time required for half of the dose to be absorbed in vivo was shorter in the ALT-SNEDDS-Water group than in the ALT-Oil group. This suggests that hydration of ALT-SNEDDS before administration accelerated oral ALT absorption. This finding agrees with previous reports showing that SNEDDS can shorten the time required to reach effective plasma exposure by improving drug dispersion and dissolution in the gastrointestinal tract. Paclitaxel-SMEDDS was reported to increase oral bioavailability by 3.8-fold and shorten the time to peak concentration by approximately 40% compared with conventional formulations [19]. In the present study, ALT-SNEDDS increased the oral bioavailability of ALT by 1.96-fold, whereas the hydrated ALT-SNEDDS-Water formulation increased it by 4.97-fold compared with ALT-Oil. Although direct comparison across different drugs and formulations should be made cautiously, the magnitude of improvement observed for ALT-SNEDDS-Water is comparable to, and even higher than, several previously reported SNEDDS-based oral delivery systems. This highlights the practical value of the hydrated SNEDDS strategy for enhancing the oral delivery of ALT.

The superior absorption of ALT-SNEDDS-Water may be explained by the factors discussed above. Rats are small and have limited gastrointestinal fluid volume; therefore, ALT-SNEDDS may not emulsify sufficiently immediately after entering the gastrointestinal tract and may require a longer emulsification time, which can affect the initial absorption rate. By contrast, pre-hydration allows the formulation to enter the gastrointestinal tract in a more fully dispersed state, thereby increasing the available surface area for drug release and absorption. This interpretation is supported by the markedly higher C_max_ and AUC values in the ALT-SNEDDS-Water group than in both the ALT-Oil and non-hydrated ALT-SNEDDS groups. Thus, while ALT-SNEDDS itself improved ALT exposure, the hydrated formulation more effectively converted the self-nanoemulsifying potential into rapid and extensive in vivo absorption.

Lymphatic transport of ALT-SNEDDS was also investigated. After lymphatic transport was blocked, the plasma ALT concentration decreased significantly, indicating that lymphatic transport is involved in ALT-SNEDDS absorption. This result is consistent with the established view that lipophilic drugs incorporated into lipid-based delivery systems can associate with intestinal lipid processing pathways and enter the lymphatic circulation through chylomicron-mediated transport [20]. Compared with direct portal absorption, lymphatic transport can avoid hepatic portal first-pass metabolism, increase drug concentration in lymphatic tissues, and indirectly improve systemic bioavailability. Therefore, the significant decrease in plasma ALT after lymphatic blockade provides mechanistic support for the enhanced bioavailability observed in the pharmacokinetic experiment.

In the oral pharmacokinetic experiment after lymphatic blockade, cycloheximide (CHX) was used as the chylomicron flow blocker. CHX is a protein synthesis inhibitor that inhibits chylomicron secretion and transport. After mature chylomicrons are synthesized, CHX prevents their secretion and release from the Golgi-associated secretory pathway by interfering with the cellular processes required for exocytosis. As a result, vesicles required for chylomicron secretion cannot form normally, preventing chylomicrons from being released and transported to the lymphatic system. However, the chylomicrons are not destroyed and can remain stored within the intracellular secretory compartment [21]. Chylomicron flow blockade has been used to study intestinal absorption, mesenteric lymph node distribution, and intestinal lymphatic uptake of self-microemulsions [22]. For example, Li et al. [23] used this approach to demonstrate that lymphatic uptake contributed to the improved bioavailability of huperzine A-loaded SMEDDS. Similarly, Manasa et al. [24] developed a Pueraria flavone-loaded self-microemulsifying drug delivery system (PF-SMEDDS), and pharmacokinetic experiments in rats showed that, before and after CHX blockade of the lymphatic transport pathway, the oral bioavailability of PF-SMEDDS was 2.57-fold and 2.28-fold higher than that of the PF suspension, respectively. This indicated that PF-SMEDDS improved oral PF absorption partly through the lymphatic absorption pathway.

Compared with these previous SMEDDS studies, the present ALT-SNEDDS study shows a similarly clear lymphatic contribution but with a stronger overall improvement in bioavailability, especially in the hydrated formulation. The 4.97-fold increase in bioavailability of ALT-SNEDDS-Water, together with the marked decrease in plasma ALT concentration after CHX treatment, suggests that lymphatic transport is not merely a theoretical pathway but a functionally relevant route for ALT-SNEDDS absorption. In summary, CHX can effectively block chylomicron-mediated lymphatic drug transport and may serve as a practical alternative to lymph duct cannulation for evaluating oral lymphatic transport because it is simpler and easier to perform.

The present study employed a rat dose of 40 mg/kg, which is substantially higher than the clinically recommended dose of ALT in the target species (pigs), approximately 0.20–0.22 mg/kg. This dose selection was primarily driven by the experimental requirements of pharmacokinetic studies maintaining detectable drug concentrations in rat plasma to obtain complete concentration time profiles, which is a routine practice in formulation development for evaluating relative bioavailability. It should be acknowledged that this high-dose strategy precludes direct extrapolation of our pharmacokinetic findings to clinical dosing scenarios.

Female Sprague-Dawley rats were chosen as the pharmacokinetic and intestinal absorption model for several reasons. First, the rat is the standard preclinical species for studying oral absorption and lymphatic transport of lipid-based formulations, providing a large body of comparable literature and well-established methodology. Second, the use of a small-animal model aligns with the principles of reduction and refinement in animal research at the early formulation-screening stage, where the aim is to evaluate the relative absorption-enhancing effect of the SNEDDS technology rather than to establish clinical dosing. Third, ALT is a progestogen whose pharmacological action depends on negative feedback at the hypothalamic–pituitary–gonadal axis; female animals were therefore selected to match the endocrine context of the target species (sows and mares) and to avoid the confounding effects of male sex hormones. It should nevertheless be acknowledged that rats differ from pigs and horses in drug-metabolizing enzymes, gastrointestinal physiology and bile secretion, and the pharmacokinetic findings cannot be directly extrapolated to the target species. To address this translational gap, a large-scale pharmacokinetic and pharmacodynamic study of ALT-SNEDDS in sows is currently underway in collaboration with Ningbo Sansheng Biotechnology Co., Ltd.; the results will be reported separately once completed.

A recent study by [25] reported the pharmacokinetics of an ALT/HP-β-CD inclusion complex in pigs at a dose of 0.31 mg/kg, with a relative bioavailability of 114.08%. Based on allometric scaling by body surface area, the porcine dose of 0.31 mg/kg corresponds to an equivalent rat dose of approximately 1.8–2.0 mg/kg. In comparison, the 40 mg/kg dose used in our study is approximately 20-fold higher than this equivalent dose. This substantial dose disparity reflects fundamental differences in experimental design and objectives between the two studies: the study [25] more closely resembles a clinical dosing scenario, whereas our study focuses on maximizing the exposure differences between formulations in a rodent model to evaluate the relative solubilization and absorption-enhancing effects of the SNEDDS technology. Importantly, the 40 mg/kg dose was applied uniformly across all experimental groups (oil solution, SNEDDS, and hydrated SNEDDS); therefore, the relative bioavailability data obtained (1.96-fold and 4.97-fold) remain reliable for inter-group comparisons. In addition to the pharmacokinetic evaluation, the safety of this high dose was assessed. A single oral administration of 40 mg/kg caused no mortality, no abnormal clinical signs, and no histopathological changes in the heart, liver, spleen, lung or kidney over a 7-day observation (Section 3.5). It should be noted, however, that this is a single-dose acute toxicity study; repeated-dose toxicity following the clinically relevant 14-day regimen in the target species (sows) remains to be addressed before clinical application.

In the intestinal perfusion experiment, an analytical method for determining ALT content in phenol red K-R buffer was successfully established, and the stability of ALT in the perfusate was evaluated and found to be good, confirming the feasibility of the method. Before the experiment, animals are generally anesthetized and surgically prepared to ensure procedural stability and animal comfort. During administration, the drug solution is perfused into the small intestinal segment. Continuous collection of the intestinal perfusate after administration allows analysis of changes in drug concentration in the intestine. By monitoring these concentration changes, the absorption rate and extent can be evaluated. This experiment provides important information about the dynamic absorption process of the drug in the intestine, including absorption rate, absorption site, and absorption amount.

However, it should be noted that various experimental factors must be strictly controlled to ensure the accuracy and reliability of the results. In rat in situ intestinal circulation experiments, excessively long circulation times or excessively high flow rates can damage the intestinal absorption environment and cause absorption results to deviate substantially from the actual physiological situation. An excessively low flow rate cannot reduce the unstirred water layer around intestinal mucosal cells, thereby reducing drug absorption [26]. Conversely, an excessively high flow rate and intestinal lumen expansion can artificially increase absorption [27]. These earlier studies emphasize that intestinal perfusion data are highly dependent on hydrodynamic conditions. Therefore, the selection of a 2 mL/min flow rate in the present study is important because it better approximates the physiological state and reduces methodological bias. In addition, because the small intestine absorbs water, correction of the circulating liquid volume and density is necessary. Since phenol red is not absorbed by the intestine, phenol red-based correction of test solution volume is crucial in rat in situ intestinal perfusion experiments. It improves reproducibility and accuracy and provides reliable data for drug absorption studies.

Because ALT has low water solubility, 1% Tween 80 was used to assist solubilization of ALT. The addition of Tween 80 may increase intestinal absorption of ALT and therefore may cause some deviation from the true absorption results of free ALT. Nevertheless, the results showed that both the absorption rate constant Ka and the effective permeability coefficient P_eff_ in the ALT group were significantly lower than those in the ALT-SNEDDS group. Specifically, ALT-SNEDDS increased K_a_ and P_eff_ by approximately 3.7-fold and 4.4-fold, respectively, compared with free ALT. This strong improvement in both absorption rate and membrane permeability provides direct intestinal-level evidence that ALT-SNEDDS enhances ALT absorption. Compared with previous ALT formulation studies that mainly focused on solubility enhancement or in vitro release, the present intestinal perfusion data provide stronger physiological evidence that SNEDDS improves not only dissolution but also intestinal permeation.

At present, two cell models, human colon adenocarcinoma cells (Caco-2) and Madin-Darby canine kidney epithelial cells (MDCKs), are widely used to study in vitro drug absorption mechanisms. The MDCK cell line is derived from Madin-Darby canine kidney epithelial cells. Because these cells have intestinal epithelial cell-like structures, numerous microvilli on the cell surface, and multiple receptor proteins expressed on the cell membrane, they are suitable for absorption and transport studies. In addition, MDCK cells grow rapidly and form a complete, compact monolayer in only 4 to 7 days, which is much shorter than the 14 to 21 days required for Caco-2 cells. Because MDCK cells are also like epithelial cells in protein composition, genetics, and cellular lipids, the MDCK cell model has increasingly been used to simulate intestinal epithelial cells in studies of absorption and transport mechanisms. Compared with studies that rely only on pharmacokinetic endpoints, the use of MDCK cells in the present work provides mechanistic evidence linking the improved in vivo absorption of ALT-SNEDDS with carrier-mediated cellular uptake.

When determining whether cellular uptake is energy dependent, sodium azide [28] can be used during incubation to inhibit the cellular respiratory system and thereby suppress the energy required for uptake. Alternatively, low temperature (4 °C) can be used to inhibit ATP-dependent cellular processes. In this study, cells incubated at 37 °C for 1 h were used as the control, and uptake of drug-loaded self-nanoemulsion by MDCK cells was examined at 4 °C. The results showed that 4 °C significantly reduced the mean fluorescence intensity in MDCK cells, indicating that cellular uptake was an energy-dependent process. This result is consistent with previous nanoparticle and self-nanoemulsion studies in which energy depletion markedly reduced cellular uptake, confirming the involvement of active internalization rather than simple passive diffusion [29]. Therefore, the cellular uptake behavior of ALT-SNEDDS supports the pharmacokinetic and intestinal perfusion findings by showing that the formulation enhances absorption through active cellular entry pathways.

Quercetin can inhibit lipid raft-mediated endocytosis [30]. Chlorpromazine is a cationic amphiphilic molecule that specifically blocks clathrin-mediated endocytosis by disrupting complexes between clathrin and the cell surface [31]. Methyl-beta-cyclodextrin can chelate cholesterol and thereby prevent caveolin-mediated endocytosis [32]. Because cholesterol is a nonpolar hydrophobic compound, the hydrophobic cavity of methyl-beta-cyclodextrin is structurally suitable for effective cholesterol chelation. Macropinocytosis is a nonspecific form of cellular endocytosis that internalizes extracellular fluid into macropinosomes, which are large irregular vesicles [33]. Macropinosomes have diameters of 0.2 to 10 μm. Their formation is not directly affected by ligands but is closely associated with the cytoskeleton [34]. Studies have shown that macropinocytosis is microtubule dependent [35]. Colchicine binds to tubulin, prevents microtubule polymerization, and inhibits dynamic microtubule stability; therefore, colchicine can inhibit macropinocytosis.

In this study, the possible uptake mechanisms of SNEDDS in MDCK cells were explored. After cells were treated with inhibitors of different endocytic pathways, uptake of SNEDDS-Cou6 was assessed by measuring intracellular mean fluorescence intensity using flow cytometry. The results indicated that the intestinal absorption mechanism of ALT-SNEDDS may involve active transport, and its cellular uptake is associated with lipid raft-, clathrin-, and caveolin-mediated endocytosis as well as macropinocytosis. This multi-pathway uptake pattern is consistent with previous reports showing that nanoscale lipid or polymeric carriers can enter epithelial cells through several endocytic routes rather than a single pathway [36]. Compared with free drug absorption, which is mainly limited by dissolution and passive membrane permeation, ALT-SNEDDS appears to enhance absorption by combining improved solubilization, lymphatic transport potential, increased intestinal permeability, and active cellular internalization. These combined mechanisms explain why ALT-SNEDDS produced higher C_max_, AUC, K_a_, and P_eff_ values than ALT-Oil or free ALT.

The present findings show that ALT-SNEDDS has a stronger and more comprehensive absorption-enhancing effect than conventional ALT formulations and is comparable to other successful SNEDDSs reported for poorly soluble drugs. More importantly, this study provides integrated evidence from pharmacokinetics, lymphatic blockade, intestinal perfusion, and cellular uptake experiments. These findings provide a reference for studying the absorption mechanism of ALT-SNEDDS in an in vitro cell model. However, the specific intracellular transport pathway after uptake remains unclear and requires further investigation.

## 5. Conclusions

Compared with the ALT oil solution, ALT-SNEDDS increased relative bioavailability by 1.96-fold in the rat pharmacokinetic experiment. Hydrated administration in rats produced an even better effect, increasing relative bioavailability by 4.97-fold, and ALT-SNEDDS showed a tendency toward lymphatic absorption. Compared with raw ALT, ALT-SNEDDS significantly improved ALT absorption throughout the small intestine in the rat in situ intestinal circulation perfusion experiment, indicating its potential to improve the oral bioavailability of ALT. ALT-SNEDDS showed low cytotoxicity to MDCK cells. Cou6 was successfully encapsulated into ALT-SNEDDS as a fluorescent indicator and could be used to indicate the in vivo uptake behavior of ALT-SNEDDS. In the MDCK monolayer cell model, ALT-SNEDDS uptake was time-dependent and energy-dependent, and the uptake pathways were associated with lipid raft-, clathrin-, and caveolin-mediated endocytosis. Moreover, a single oral dose of 40 mg/kg did not induce acute toxicity or histopathological changes in the major organs of rats.

The ALT-SNEDDS constructed in this study improves the oral bioavailability of ALT and provides a foundation for subsequent formulation development. This study examined the plasma concentration of ALT-SNEDDS after oral administration, the cellular uptake mechanism in an in vitro cell model, and acute oral toxicity in rats. Further pharmacokinetic and pharmacodynamic studies in the target species (sows) at clinically relevant doses are needed to evaluate the translational potential of this formulation.

## Figures and Tables

**Table 1:** Types and concentrations of endocytosis inhibitors.

Category	Endocytic Pathway	Dose
Quercetin	Lipid raft-mediated endocytosis	20 μg/mL
Methyl-β-cyclodextrin	Caveolin-mediated endocytosis	10 mmol/L
Chlorpromazine	Clathrin-mediated endocytosis	30 μmol/L
Colchicine	Macropinocytosis	1 μmol/L

**Table 2:** Pharmacokinetic parameters in rats after oral administration of three different formulations (Mean ± SD) (n = 5). Statistical significance was determined using two-way ANOVA followed by Tukey’s post-hoc test.

Pharmacokinetic Parameter	ALT-Oil	ALT-SNEDDS	ALT-SNEDDS-Water
T_1/2Ka_ (h)	0.32 ± 0.31	0.43 ± 0.24	0.2 ± 0.03
T_1/2_z (h)	2.56 ± 0.67	3.15 ± 0.51	2.85 ± 0.08
C_max_ (μg/mL)	0.87 ± 0.21	1.33 ± 0.49 *	6.83 ± 1.06 **^#^
K_a_ (1/h)	3.33 ± 1.78	3.51 ± 0.53	2.11 ± 1.20
T_max_ (h)	0.77 ± 0.38	0.67 ± 0.39	0.50 ± 0.02
AUC_(0-t)_ (μg/mL·h)	2.62 ± 0.94	5.41 ± 1.89 *	13.03 ± 1.42 **^#^
AUC_(0-∞)_ (μg/mL·h)	2.93 ± 1.04	6.14 ± 1.84 *	13.97 ± 1.18 **^#^
MRT_(0-t)_ (h)	2.62 ± 0.64	3.22 ± 0.87	2.79 ± 0.39
MRT_(0-∞)_ (h)	4.14 ± 1.50	3.73 ± 1.04	3.57 ± 0.13
CLz/F (L/h/kg)	14.81 ± 4.17	7.01 ± 1.15 *	2.88 ± 0.23 **^#^
Vz/F (L/kg)	53.67 ± 8.02	31.83 ± 6.93 **	11.83 ± 1.67 **^#^

Note: Compared with ALT-Oil, * *p* < 0.05, ** *p* < 0.01; compared with ALT-SNEDDS, ^#^ *p* < 0.01.

**Table 3:** Pharmacokinetic parameters of ALT-SNEDDS-Water after direct oral administration and after intraperitoneal CHX injection in rats (Mean ± SD) (n = 5). Statistical significance was determined using two-way ANOVA followed by Tukey’s post-hoc test.

Pharmacokinetic Parameter	SNEDDS-Water	SNEDDS-Water-CHX
T_1/2z_ (h)	2.85 ± 0.08	4.12 ± 2.19
C_max_ (μg/mL)	6.83 ± 1.06	2.95 ± 0.49 *
T_max_ (h)	0.50 ± 0.02	0.38 ± 0.14
AUC_(0-2h)_ (μg/mL·h)	6.65 ± 0.38	3.79 ± 0.80 **
AUC_(0-12h)_ (μg/mL·h)	13.03 ± 1.42	10.39 ± 1.38
MRT_(0-t)_ (h)	2.79 ± 0.39	2.62 ± 0.64
CL/F (L/h/kg)	2.73 ± 0.22	3.43 ± 0.78

Note: Compared with SNEDDS-Water, *, *p* < 0.05, **, *p* < 0.01.

## Data Availability

The original contributions presented in the study are included in the article. Further inquiries can be directed to the corresponding author.

## Supplementary Materials

The following supporting information can be downloaded at: https://www.mdpi.com/article/10.3390/pharmaceutics18101336/s1, Table S1 Solubility of ALT in different solvents; Table S2 Mutual solubility between candidate oil phases, surfactants and co-surfactants; Table S3 Independent variables and their levels used in the central composite design; Table S4 Central composite design matrix and experimental responses for ALT-SNEDDS optimisation; Table S5 ANOVA for the quadratic model of self-emulsifying time (*Y*_1_); Table S6 ANOVA for the quadratic model of droplet size (*Y*_2_); Table S7 Validation of the CCD-RSM-predicted optimum; Table S8 Drug content of ALT-SNEDDS stored at 4 °C, 25 °C and 60 °C (mean ± SD, n = 3); Table S9 Appearance of ALT-SNEDDS stored at 4 °C, 25 °C and 60 °C; Table S10 Encapsulation efficiency of ALT-SNEDDS (n = 3); Table S11 Regression equation of ALT-SNEDDS release for reverse dialysis (reverse dialysis, PBS + 2 wt% Tween 80); Table S12 Regression equation of ALT-Oil release for reverse dialysis (reverse dialysis, PBS + 2 wt% Tween 80); Table S13 Results of intra-day and inter-day precision (n = 3); Table S14 Results of the recovery rate (n = 3); Table S15 Results of intraday and interday precision (n = 3); Table S16 Results of the recovery rate (n =3 ); Table S17 Organ coefficients (%) of rats (mean ± SD); Figure S1 Pseudo-ternary phase diagrams of ALT-SNEDDS with different oil phases. (a) Oleic acid (OC); (b) Ethyl oleate (EO); (c) OC: EO = 1:1 (m/m). Surfactant-to-co-surfactant mass ratio (*K_m_*) fixed at 1:1. Region A = self-nanoemulsion region; Region B = nanoemulsion region; Figure S2 Pseudo-ternary phase diagrams of ALT-SNEDDS at different K_m_ values (Tween 80: Transcutol HP). (a) K_m_ = 1:2; (b) Km = 1:1; (c) K_m_ = 2:1; (d) K_m_ = 3:1. EO used as the oil phase. Region A = self-nanoemulsion region; Region B = nanoemulsion region; Figure S3 Three-dimensional response surface plots of (a) self-emulsifying time and (b) mean droplet size as functions of oil mass fraction (*X*_1_) and Km (*X*_2_); Figure S4 Preparation of ALT-SNEDDS; Figure S5 Appearance of the optimised ALT-SNEDDS. (A) Blank SNEDDS; (B) ALT-SNEDDS; (C) aqueous dispersion of ALT-SNEDDS (1:100); (D) showing the Tyndall effect when irradiated with a red laser pointer; Figure S6 Results of nanoemulsion type identification of ALT-SNEDDS; Figure S7 (A) Particle size distribution and (B) zeta potential of the ALT-SNEDDS nanoemulsion; Figure S8 Transmission electron micrographs of ALT-SNEDDS nanoemulsion droplets, stained with 2 wt% phosphotungstic acid. Scale bars: (A) 50 nm; (B) 100 nm. Magnification: 50,000×; accelerating voltage: 80 kV; Figure S9 Effect of aqueous dilution on the droplet size of ALT-SNEDDS (n = 3); Figure S10 Cumulative release curves of ALT-SNEDDS and ALT-Oil for dialysis; Figure S11 Cumulative release curves of ALT-SNEDDS and ALT-Oil for reverse dialysis; Figure S12 HPLC images of blank plasma samples; Figure S13 HPLC images of blank plasma samples of ALT standard; Figure S14 HPLC images of Plasma samples from rats after oral administration of ALT; Figure S15 Standard curve for ALT in plasma; Figure S16 Standard curve of phenol red in phenol red /K-R buffer; Figure S17 HPLC chromatogram of blank (A) / ALT (B) in phenol red /K-R perfusion and enteral irrigation fluid (C); Figure S18 Standard curve of ALT in Phenol Red-KR Buffer; Figure S19 Investigation of ALT stability in internal perfusion fluid in 24 h (n = 3); Figure S20 Body weight (g) of rats in each group during the 7-day observation period (mean ± SD); Figure S21 Representative H&E-stained sections of the heart, liver, spleen, lung and kidney from each group.

## Abbreviations

The following abbreviations are used in this manuscript:

ALT	Altrenogest
SMEDDS	Self-microemulsifying drug delivery systems
MDCK	Madin-Darby canine kidney (epithelial cells)
C_max_	Peak concentration/maximum blood concentration
AUC	Area under the drug concentration–time curve
K_a_	Absorption rate constant
P_eff_	Effective permeability/effective permeability coefficient
GnRH	Gonadotropin-releasing hormone
FSH	Follicle-stimulating hormone
LH	Luteinizing hormone
BCS	Biopharmaceutics Classification System
SBA-15	Mesoporous silica SBA-15
K-R/KR powder/buffer	Krebs-Ringer powder/buffer
Cou6	Coumarin 6
CHX	Cycloheximide
EO	Ethyl oleate
PBS	Phosphate-buffered saline
DMEM	Dulbecco’s Modified Eagle Medium
FBS	Fetal bovine serum
EDTA	Ethylenediaminetetraacetic acid
CCK-8	Cell Counting Kit-8
DMSO	Dimethyl sulfoxide
SD rats	Sprague-Dawley rats
EP tube	Eppendorf tube
E_x_/E_m_	Excitation/emission wavelengths
DAPI	4′,6-diamidino-2-phenylindole
HPLC	High-performance liquid chromatography
MRT	Mean residence time
CLz/F or CL/F	Clearance
Vz/F	Volume of distribution
MFI	Mean fluorescence intensity
ATP	Adenosine triphosphate
Caco-2	Human colon adenocarcinoma cells
PF	Pueraria flavone
