# Supplementary material for: Enhanced Oral Bioavailability and Pharmacokinetics of Altrenogest via a Self-Nanoemulsifying Drug Delivery System
Source: Pharmaceutics. 2026 Oct 21;18(10):1336. doi: 10.3390/pharmaceutics18101336 (PMC13708577; doi:10.3390/pharmaceutics18101336)
Supplement: Supplementary file 1 [file pharmaceutics-18-01336-s001.zip › pharmaceutics-4597256-supplementary.pdf]

Supplementary Materials

S1 Formulation Screening

S1.1 Equilibrium solubility of ALT in candidate excipients

An excess of ALT raw material was added to a sealed glass vial containing 3 mL of each candidate oil, surfactant or co-surfactant. The vials were shaken at 100 rpm in a 37 °C water bath for 24 h and then equilibrated without agitation for a further 24 h. The supernatant was diluted with acetonitrile, filtered through a 0.22 μm membrane and analysed by HPLC. Each measurement was performed in triplicate.

ALT showed the highest solubility in Transcutol HP (38.05 mg/mL), followed by Tween 80 (32.96 mg/mL) and EO (25.46 mg/mL). Solubility in water was 0.04 mg/mL at 25 °C. These three excipients were therefore selected for the compatibility screen, along with OC, GTCC, Maisine CC, Labrafac MC60, Labrasol and Capryol 90 for parallel comparison.

Table S1 Solubility of ALT in different solvents

Solvent / Excipient	Solubility (mg/g)
n-Butanol	306.72
n-Propanol	269.99
Isopropanol	237.32
Ethyl acetate	168.10
n-Octanol	91.34
1,2-Propanediol (Propylene glycol)	78.31
Oleic acid	68.98
Polyethylene glycol 400 (PEG 400)	48.79
Diethylene glycol monoethyl ether (Transcutol HP)	38.55
Propylene glycol monocaprylate (Capryol 90)	39.64
Glyceryl tricaprylate/caprate (GTCC)	35.89
Polysorbate 80 (Tween 80)	29.96
Caprylic/capric mono- and di-glycerides (Labrafac MC60)	32.34
Propylene glycol dicaprylocaprate (Labrafac PG)	32.77
Glyceryl monolinoleate (Maisine CC)	29.25
Ethyl oleate (EO)	29.26
Caprylocaproyl polyoxyl-8 glycerides (Labrasol)	23.51
Isopropyl myristate (IPM)	26.47

S1.2 Excipient compatibility

Oil : surfactant : co-surfactant combinations (4:3:3 by mass, total 1 g) were vortex-mixed for 30 s

and then kept at room temperature for 30 min. Miscibility was scored as clear (√) or phase-separated (×). For miscible systems, 0.5 g of the blend was diluted with 5 mL of ultrapure water, vortex-mixed and kept at room temperature for 24 h; phase separation was recorded as absent (√) or present (×). Aqueous dispersions were then graded on a five-point self-microemulsion scale (Table S2).

Table S2 Mutual solubility between candidate oil phases, surfactants and co-surfactants (4:3:3, m/m; total 1 g)

Oil phase	Surfactant	Co-surfactant	Miscible	No phase separation
Maisine CC	Tween 80	Capryol 90	√	×
Maisine CC	Tween 80	Transcutol HP	√	×
Maisine CC	Labrafac MC60	Capryol 90	×	√
Maisine CC	Labrafac MC60	Transcutol HP	×	√
Maisine CC	Labrasol	Capryol 90	√	×
Maisine CC	Labrasol	Transcutol HP	√	×
OC	Tween 80	Capryol 90	√	×
OC	Tween 80	Transcutol HP	√	√
OC	Labrafac MC60	Capryol 90	√	×
OC	Labrafac MC60	Transcutol HP	√	×
OC	Labrasol	Capryol 90	√	×
OC	Labrasol	Transcutol HP	√	×
EO	Tween 80	Capryol 90	√	√
EO	Tween 80	Transcutol HP	√	√
EO	Labrafac MC60	Capryol 90	×	√
EO	Labrafac MC60	Transcutol HP	×	√
EO	Labrasol	Capryol 90	×	×
EO	Labrasol	Transcutol HP	×	×

S1.3 Construction of pseudo-ternary phase diagrams

Oil and mixed surfactant/co-surfactant were combined at mass ratios ranging from 1:9 to 9:1 to give

nine pre-mixtures. Ultrapure water was added dropwise at 100 rpm under magnetic stirring at 37 °C, and the mass of water added was recorded until the dispersion turned turbid. For each titration point, the dispersion was classified as clear, slightly bluish, milky or phase-separated; clear or bluish dispersions exhibiting the Tyndall effect with a red laser pointer were classified as nanoemulsions. The self-nanoemulsion region (region A) was defined as the nanoemulsion region that could be diluted infinitely with water, identified by drawing an extension line from the first nanoemulsion point towards the mixed-surfactant apex. Pseudo-ternary phase diagrams were plotted with OriginPro 2018, using water, oil and mixed surfactant as the three vertices.

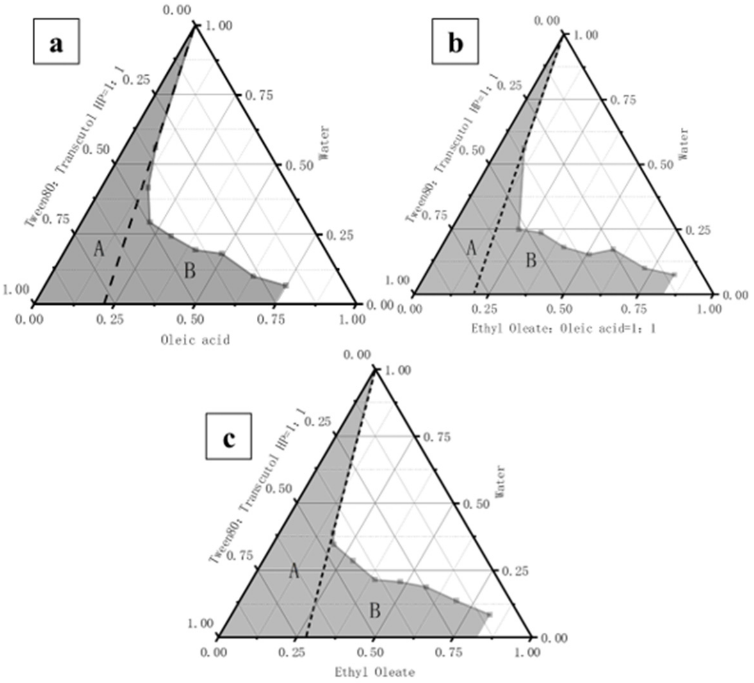

Figure S1 Pseudo-ternary phase diagrams of ALT-SNEDDS with different oil phases. (a) Oleic acid (OC); (b) Ethyl oleate (EO); (c) OC : EO = 1:1 (m/m). Surfactant-to-co-surfactant mass ratio (K_m) fixed at 1:1. Region A = self-nanoemulsion region; Region B = nanoemulsion region.

S1.4 Effect of oil phase and surfactant-to-co-surfactant ratio (K_m)

Pseudo-ternary phase diagrams were first constructed with the surfactant and co-surfactant mass ratio fixed at 1:1, while OC, EO and OC:EO = 1:1 (m/m) were screened as the oil phase. The oil phase producing the largest self-nanoemulsion region was then selected. Using this oil, four K_m values (Tween 80 : Transcutol HP = 1:2, 1:1, 2:1, 3:1) were screened; the diagram with the largest self-nanoemulsion region while avoiding a gel-like phase was chosen to define the working ranges

for the central composite design.

EO gave the largest self-nanoemulsion area at K_m = 1:1, larger than those of OC and OC:EO = 1:1, and was therefore selected as the oil phase. Increasing K_m progressively enlarged the self-nanoemulsion region; at K_m = 3:1, however, a gel-like phase appeared during titration. Considering the nanoemulsion area, surfactant safety, drug solubility and gel formation, the working ranges were set to 20.0–35.0 wt% for the oil fraction and 1.0–2.5 for K_m.

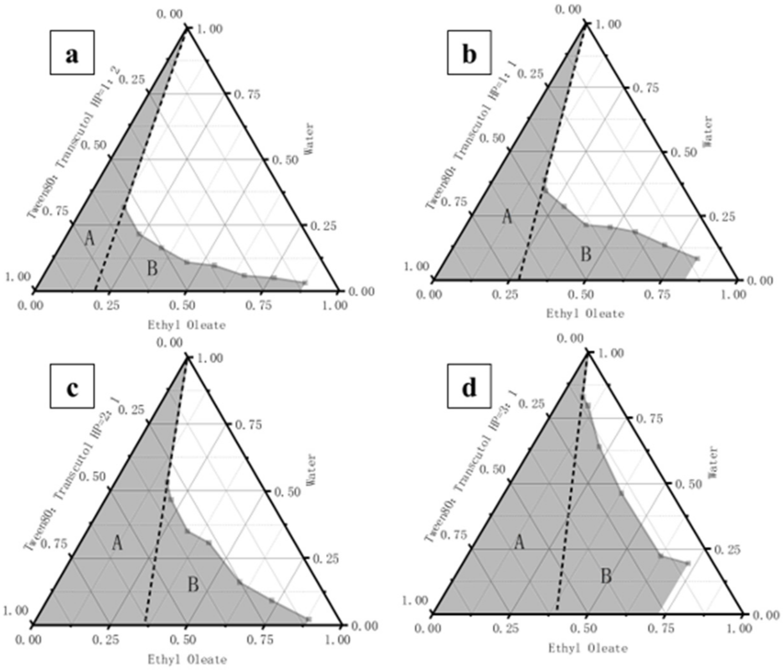

Figure S2 Pseudo-ternary phase diagrams of ALT-SNEDDS at different K_m values (Tween 80 : Transcutol HP). (a) K_m = 1:2; (b) K_m = 1:1; (c) K_m = 2:1; (d) K_m = 3:1. EO used as the oil phase. Region A = self-nanoemulsion region; Region B = nanoemulsion region.

S1.5 Central composite design–response surface methodology (CCD-RSM)

A two-factor CCD with five levels (−α, −1, 0, +1, +α; α = 1.414) was generated with Design-Expert 10.0, using oil mass fraction (X_1, 20.0–35.0 wt%) and K_m (X_2, 1.0–2.5) as independent variables. Self-emulsifying time (Y_1, s) and mean droplet size (Y_2, nm) were used as responses. Five replicate centre points were included to enable estimation of pure error. Thirteen formulations were prepared and characterised. Responses were fitted to linear and quadratic models by multiple regression, and model adequacy was assessed by ANOVA, lack-of-fit test and coefficient of determination (R^2). Three-dimensional response surface plots and desirability optimisation were used to identify the

optimal composition.

Table S3 Independent variables and their levels used in the central composite design

Factors	Levels				
	-α	-1	0	1	+α
Oil% (X_1, mass fraction)	0.2	0.22	0.28	0.33	0.35
K_m (X_2, Tween 80 : Transcutol HP)	1	1.22	1.75	2.28	2.5

Table S4 Central composite design matrix and experimental responses for ALT-SNEDDS optimisation.

No.	X_1 (Oil %)	X_2 (K_m)	Self-emulsifying time Y_1 (s)	Droplet size Y_2 (nm)
1	0.22	1.22	8.32	43.22
2	0.33	1.22	8.12	116.43
3	0.22	2.28	35.98	19.64
4	0.33	2.28	43.84	89.70
5	0.20	1.75	18.97	25.04
6	0.35	1.75	30.70	138.63
7	0.28	1.00	3.95	65.67
8	0.28	2.50	50.94	25.91
9	0.28	1.75	41.86	52.29
10	0.28	1.75	35.31	51.29
11	0.28	1.75	37.40	52.08
12	0.28	1.75	36.90	47.86
13	0.28	1.75	33.11	51.17

Table S5 ANOVA for the quadratic model of self-emulsifying time (Y_1)

Source	Sum of square	*Df*	Mean square	*F* value	*P* value	Significance
Model	2633.07	5	526.61	59.96	<0.0001	**
X_1	73.57	1	73.57	8.38	0.0232	*
X_2	2106.95	1	2106.95	239.90	<0.0001	**
X_1X_2	16.27	1	16.27	1.85	0.2157	
X_1^2	299.33	1	299.33	34.08	0.0006	**
X_2^2	192.08	1	192.08	21.87	0.0023	**
Residual	61.48	7	8.78			
Lack of Fit	19.74	3	6.58	0.63	0.6329	not significant
Pure error	41.74	4	10.44			
Cor total	2694.55	12				

*Note: ** indicates extremely significant (P< 0.01); * indicates significant (P< 0.05);* R^2=0.9772; R_{adj}^2=0.9609

Table S6 ANOVA for the quadratic model of droplet size (Y_2)

Source	Sum of square	*Df*	Mean square	*F* value	*P* value	Significance
Model	14904.38	5	2980.88	254.41	<0.0001	**
X_1	11546.38	1	11546.38	985.47	<0.0001	*
X_2	1418.89	1	1418.89	121.10	<0.0001	**
X_1 X_2	2.48	1	2.48	0.21	0.6594	
X_1^2	1827.33	1	1827.33	155.96	<0.0001	**
X_2^2	22.94	1	22.94	1.96	0.2045	
Residual	82.02	7	11.72			
Lack of fit	65.70	3	21.90	5.37	0.0691	not significant
Pure error	16.32	4	4.08			
Cor total	14986.39	12				

*Note: ** indicates extremely significant (P< 0.01); * indicates significant (P< 0.05);* R^2=0.9945; R_{adj}^2=0.9906

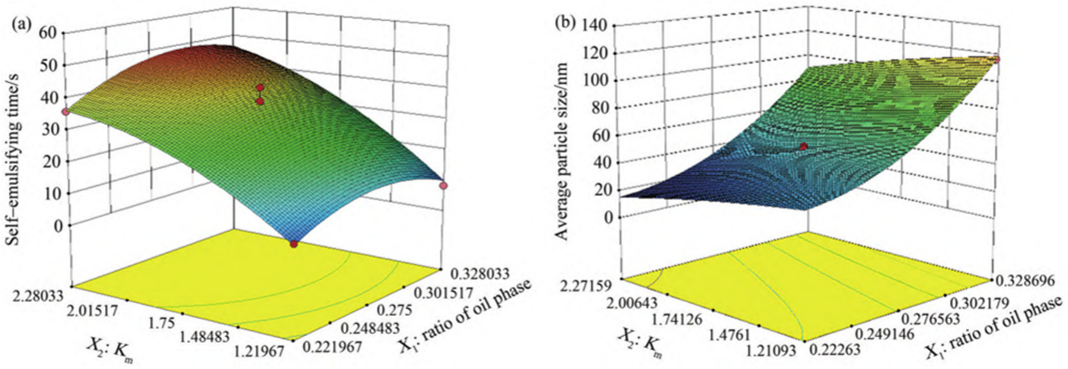

Figure S3 Three-dimensional response surface plots of (a) self-emulsifying time and (b) mean droplet size as functions of oil mass fraction (X_1) and K_m (X_2).

Table S7 Validation of the CCD-RSM-predicted optimum (EO 22.0 %, Tween 80 54.2 %, Transcutol HP 23.8 %); mean ± SD, n = 3.

Response	Predicted	Observed	Bias (%)
Self-emulsifying time Y_1 (s)	36.28	35.76 ± 1.10	−1.43
Droplet size Y_2 (nm)	17.13	18.39 ± 0.03	+7.36

S2 Preparation of ALT-SNEDDS

The optimal blank SNEDDS was prepared by weighing EO (22.0 wt%), Tween 80 (54.2 wt%) and Transcutol HP (23.8 wt%) into a sealed vial and vortexing for 30 s until a clear, transparent, isotropic mixture was obtained. The model drug was added at a drug loading of 20 mg/g and dissolved by shaking in a 37 °C water bath until a clear ALT-SNEDDS was obtained. The product was stored in sealed amber vials at room temperature.

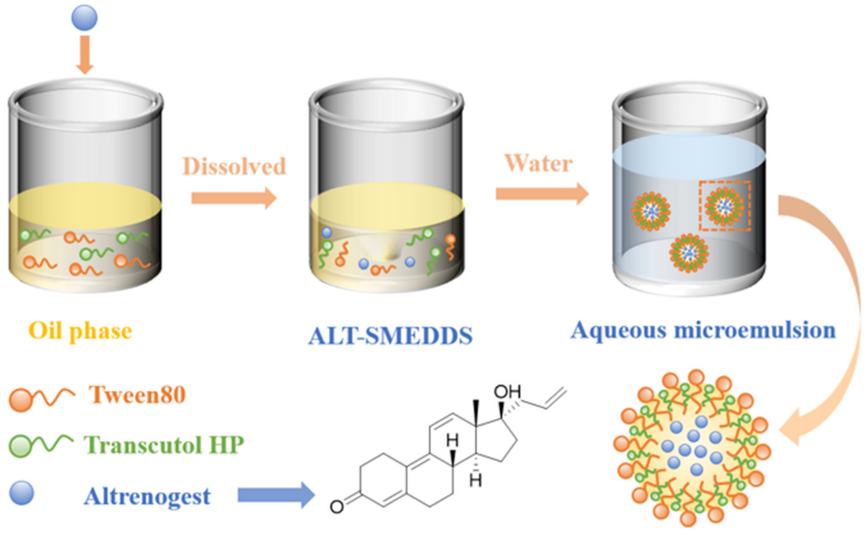

Figure S4 Preparation of ALT-SNEDDS

Note: Since this figure was created during earlier research, the term "SNEDDS" in the figure is represented as "SMEDDS."

S3 Physicochemical characterisation of ALT-SNEDDS

S3.1 Self-emulsifying time and appearance

The self-emulsifying time was determined by adding 0.1 g of ALT-SNEDDS to 10 mL of ultrapure water at 37 °C under magnetic stirring at 50 rpm. Timing began when the formulation touched the water surface and stopped when the dispersion became completely clear with no visible lumps.

Using the optimal formulation, the self-emulsifying time was 35.76 ± 1.10 s (n = 3).

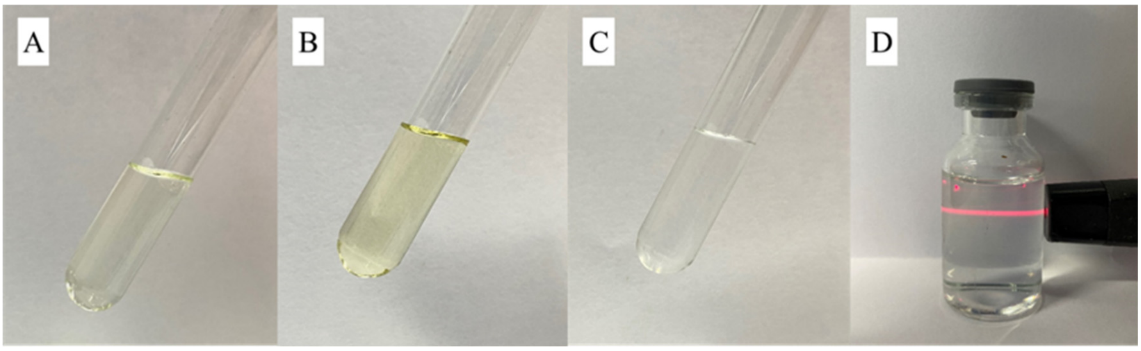

Figure S5 Appearance of the optimised ALT-SNEDDS. (A) Blank SNEDDS; (B) ALT-SNEDDS; (C) aqueous dispersion of ALT-SNEDDS (1:100); (D) showing the Tyndall effect when irradiated with a red laser pointer.

S3.2 Nanoemulsion type

The type of nanoemulsion (O/W versus W/O) was determined by the dye-diffusion method. Equal volumes of a water-soluble dye (methylene blue) and an oil-soluble dye (Sudan III) were added separately to freshly prepared ALT-SNEDDS nanoemulsion, and the diffusion rate of each dye was observed visually.

The dye-diffusion test showed rapid spreading of methylene blue but not of Sudan III, indicating that the nanoemulsion is of the O/W type.

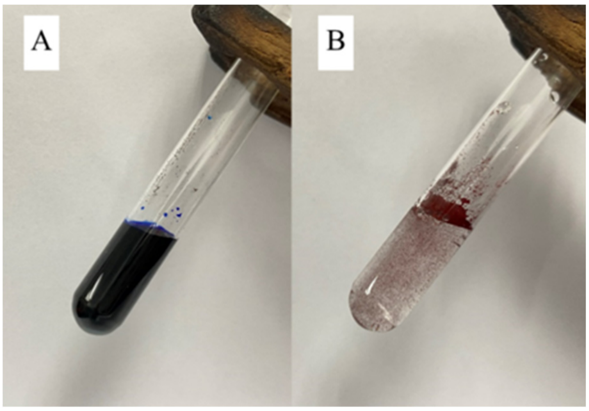

Figure S6 Results of nanoemulsion type identification of ALT-SNEDDS

S3.3 Particle size, PDI and zeta potential

Three batches of ALT-SNEDDS were diluted 100-fold with ultrapure water (i.e. 1 g ALT-SNEDDS in 100 mL ultrapure water) and equilibrated at 37 °C for 2 min. Particle size and polydispersity index (PDI) were measured by dynamic light scattering (DLS), and zeta potential was determined by electrophoretic light scattering (ELS) on a Malvern Zetasizer Nano ZS. The autocorrelation function was accumulated for 30 s and data were processed with the CONTIN algorithm.

The mean droplet size was 18.39 ± 0.03 nm with a PDI of 0.083 ± 0.090 and a zeta potential of −1.12 ± 0.12 mV (n = 3), confirming a narrow, monodisperse, near-neutral nanoemulsion.

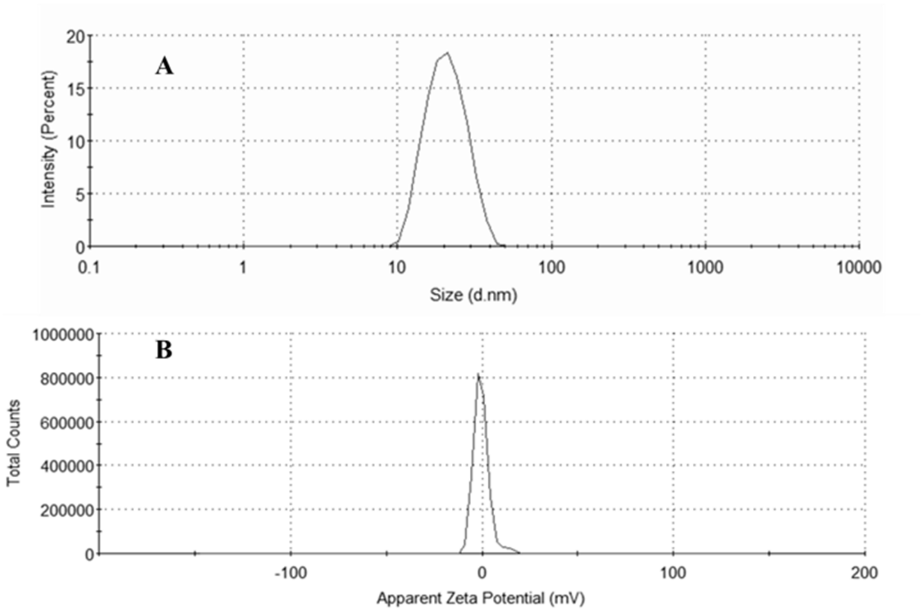

Figure S7 (A) Particle size distribution and (B) zeta potential of the ALT-SNEDDS nanoemulsion (1:100 dilution in ultrapure water, n = 3).

S3.4 Transmission electron microscopy (TEM)

A drop of the diluted nanoemulsion was placed on a carbon-coated copper grid for 1 min, negatively stained with 2 wt% phosphotungstic acid for 30 s and air-dried. Micrographs were acquired on a JEOL JEM-1400FLASH instrument at an accelerating voltage of 80 kV and a magnification of 50 000×.

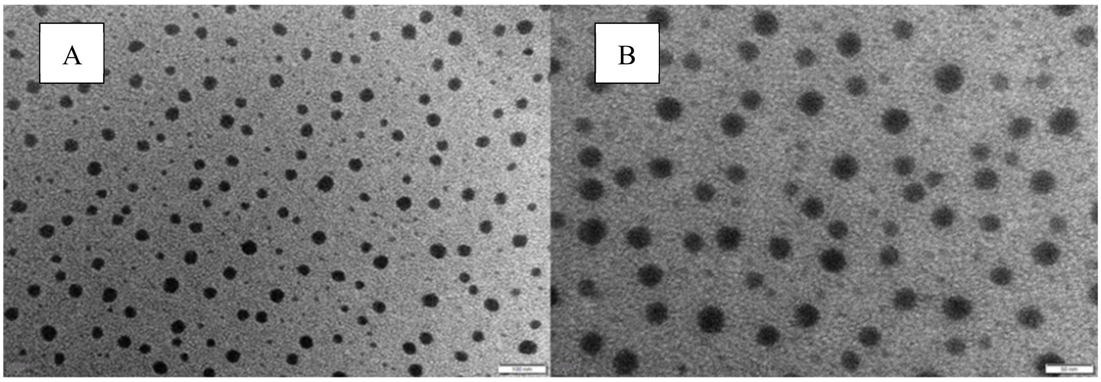

Figure S8 Transmission electron micrographs of ALT-SNEDDS nanoemulsion droplets, stained with 2 wt% phosphotungstic acid. Scale bars: (A) 50 nm; (B) 100 nm. Magnification: 50 000×; accelerating voltage: 80 kV.

S3.5 Dilution stability

ALT-SNEDDS was diluted with ultrapure water to 5, 10, 20, 50, 100, 500 and 1 000 times its original volume. After equilibration at room temperature for 12 h, each dilution was examined visually for phase separation, turbidity or drug precipitation, and droplet size was measured.

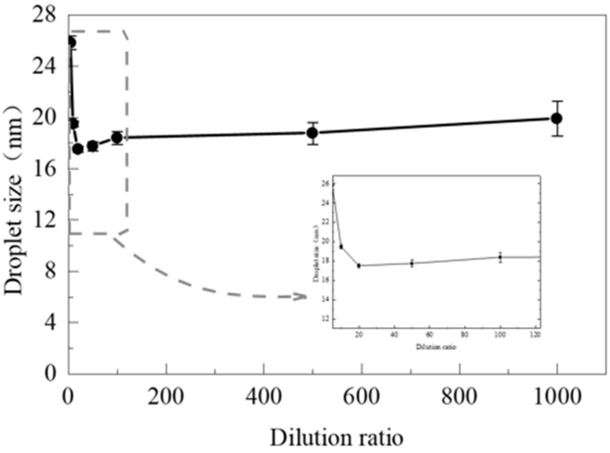

Figure S9 Effect of aqueous dilution on the droplet size of ALT-SNEDDS (n = 3)

S3.6 Temperature stability

Equal volumes of ALT-SNEDDS were sealed in glass vials and stored in the dark at 4 °C, 25 °C and 60 °C. Samples were withdrawn on day 0, 5 and 10. The drug content was quantified by HPLC after acetonitrile dilution, and the appearance (colour, clarity) was recorded photographically.

No significant change in drug content or appearance was observed at 4 °C or 25 °C over 10 days. At 60 °C the colour deepened and the drug content fell to 46 % of the initial value by day 10,

indicating that the formulation should be stored below 25 °C and protected from oxygen.

Table S8 Drug content of ALT-SNEDDS stored at 4 °C, 25 °C and 60 °C (mean ± SD, n = 3)

	4℃（%）	25℃（%）	60℃（%）
5d	99.54±1.79	99.16±3.65	61.37±2.20
10d	99.33±2.63	96.54±0.67	46.10±0.25

Table S9 Appearance of ALT-SNEDDS stored at 4 °C, 25 °C and 60 °C

**Time (d)**	**4 °C**	**25 °C**	**60 °C**
0 d	Light yellow, clear	Light yellow, clear	Light yellow, clear
5 d	Light yellow, clear	Light yellow, clear	Dark yellow, clear
10 d	Light yellow, clear	Light yellow, clear	Brown, clear

S3.7 Drug loading and encapsulation efficiency

ALT-SNEDDS (0.1 g, accurately weighed) was diluted 100-fold with acetonitrile, sonicated for 30 min and filtered through a 0.22 μm membrane. The drug content (W_1) was quantified by HPLC. A second 0.1 g sample was diluted 10-fold with purified water and centrifuged at 4000 × g for 10 min. One mL of the supernatant was diluted 10-fold with acetonitrile and the drug content (W_2) was determined. The encapsulation efficiency (EE) was calculated as EE (%) = W_2 / W_1 × 100. Measurements were performed in triplicate.

Mean drug loading = 19.44 ± 0.39 mg/g; mean EE = 98.85 ± 0.69 %.

Table S10 Encapsulation efficiency of ALT-SNEDDS (n = 3)

**No.**	**Pre-centrifugal (μg/mL)**	**Post-centrifugal (μg/mL)**	**EE (%)**
1	30.04	29.88	99.47
2	35.02	34.35	98.10
3	31.01	30.69	98.97

S3.8 In vitro drug release

Drug release from ALT-SNEDDS was evaluated against an ALT-oil solution (10 mg/mL in glyceryl tricaprylate/caprate) using a dialysis method (MWCO 7 000 Da). PBS containing 2 wt% Tween 80 was used as the release medium to maintain sink conditions. For the forward-dialysis study, 1 mL of each formulation (1 mg/mL) was sealed in a dialysis bag and immersed in 49 mL of release medium at 37 °C with shaking at 100 rpm. At predetermined time points (5, 15, 30 min, 1, 2, 4, 6, 8, 12, 24, 48 h), 1 mL of the medium was withdrawn and replaced with an equal volume of fresh medium. For the reverse-dialysis study, eight dialysis bags each containing 2 mL of release medium were equilibrated in 800 mL of medium for 10 min; 1 mL of ALT-SNEDDS or ALT-oil was then added to the external medium, and one bag was withdrawn at each sampling point. Drug concentrations were quantified by HPLC and cumulative release was calculated with correction for medium replacement. Release profiles were fitted to zero-order, first-order, Higuchi and Ritger–Peppas models using OriginPro 2018.

By forward dialysis, ALT-SNEDDS released 47.96 % of its load within 4 h, 74.77 % within 12 h and 91.41 % within 48 h, whereas the ALT-oil solution released 13.68 % and 32.42 % at the corresponding time points. By reverse dialysis, ALT-SNEDDS released > 90 % of its load within 12 h, consistent with rapid emulsification and dissolution upon contact with the aqueous medium. Fitting of the reverse-dialysis data indicated that ALT-SNEDDS release followed first-order kinetics (R^2 = 0.9473), whereas ALT-oil release was best described by the Higuchi model (R^2 = 0.9943).

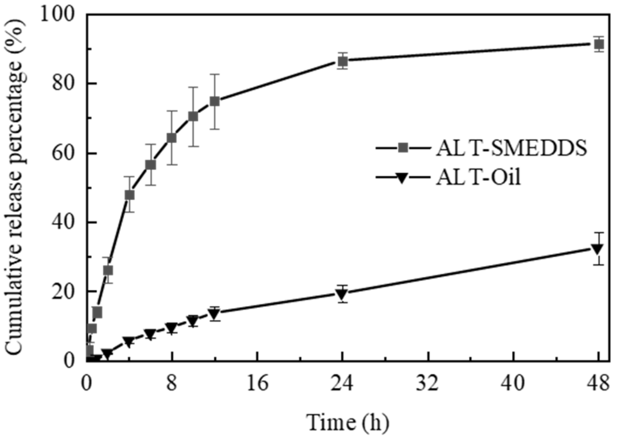

Figure S10 Cumulative release curves of ALT-SNEDDS and ALT-Oil for dialysis

Note: Since this figure was created during earlier research, the term “SNEDDS” in the figure is

represented as "SMEDDS."

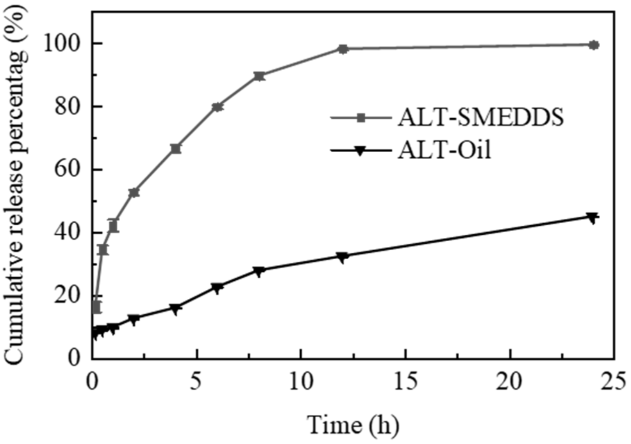

Figure S11 Cumulative release curves of ALT-SNEDDS and ALT-Oil for reverse dialysis

Note: Since this figure was created during earlier research, the term "SNEDDS" in the figure is represented as "SMEDDS."

Table S11 Regression equation of ALT-SNEDDS release for reverse dialysis (reverse dialysis, PBS + 2 wt% Tween 80)

**Model**	**Regression equation**	**R^2**
Zero-order	Q =0.0133t+0.6831	0.5217
First-order	Q=0.9943*(1-$e^{-1.3173t}$)	0.9473
Higuchi	Q=0.1033$t^{1/2}$+0.4997	0.6958
Ritger-peppas	Q=0.5533$t^{0.1874}$	0.8289

Table S12 Regression equation of ALT-Oil release for reverse dialysis (reverse dialysis, PBS + 2 wt% Tween 80)

**Model**	**Regression equation**	**R^2**
Zero-order	Q =0.0133t+0.1427	0.9016
First-order	Q =0.4850*(1-$e^{-0.1064t}$)	0.9436
Higuchi	Q =0.0837$t^{1/2}$+0.0388	0.9943
Ritger-peppas	Q =0.1142$t^{0.4285}$	0.9882

S4 Development of the HPLC Method for Determining the Concentration of Altrenogest in Plasma

S4.1 HPLC Chromatographic Conditions

Chromatography column: Kromasil C18 (150 mm × 4.6 mm, 5 μm); mobile phase: acetonitrile–water (60:40); detection wavelength: 346 nm; flow rate: 1.0 mL·min^{-1}; injection volume: 20 μL.

S4.2 Processing of rat plasma samples

A 0.2 mL plasma sample was transferred to an EP tube, and 0.8 mL ethyl acetate was added. The mixture was vortexed for 3 min and centrifuged at 8200 xg for 10 min at 4°C. Then, 0.7 mL of the upper clear layer was transferred to another clean centrifuge tube. Another 0.8 mL ethyl acetate was added to the tube containing the remaining plasma residue, and the above extraction procedure was repeated. The extraction was performed three times in total, and the combined extracts were vacuum-dried. After complete evaporation, 0.1 mL acetonitrile was added to the tube, followed by vortexing for 2 min and ultrasonication for 3 min to redissolve the residue. The sample was centrifuged at 11800 xg for 10 min, and the upper clear layer was filtered through a 0.22 μm polytetrafluoroethylene syringe filter. A 10 μL aliquot of the filtrate was injected for HPLC analysis.

S4.3 Experiment on Method Specificity

Blank plasma samples, ALT standard solutions, and plasma samples collected from rats after drug administration were injected separately to investigate whether endogenous impurities interfered with the retention time of ALT. The results show that the peak elution time for ALT is approximately 5.1 min. There was no interference from endogenous substances near the peak elution time, and the baseline remained stable, indicating high specificity; therefore, the detection method is feasible.

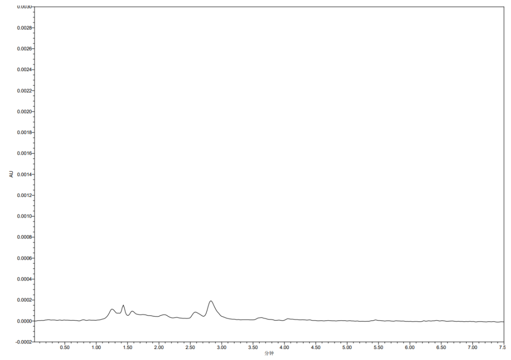

Figure S12 HPLC images of blank plasma samples

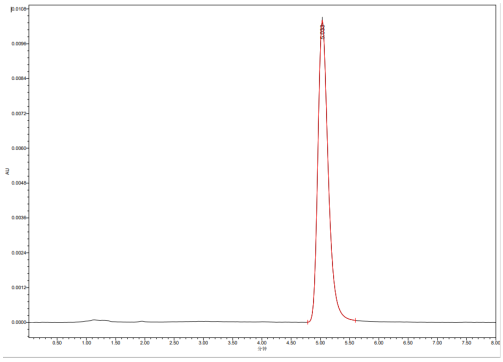

Figure S13 HPLC images of blank plasma samples of ALT standard

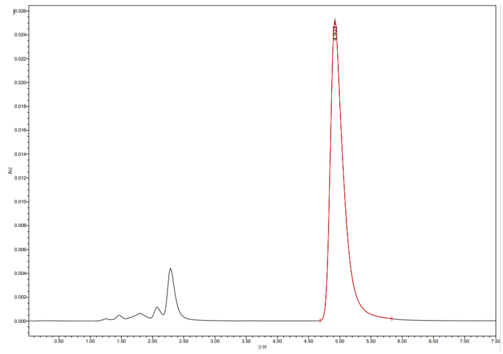

Figure S14 HPLC images of Plasma samples from rats after oral administration of ALT

S4.4 Determination of the Limit of Detection (LOD), the Limit of Quantification (LOQ) and Standard curve

The standard curve is shown in Figure S15. The regression equation is: $A = 61,481C - 543.39$ ($R^2 = 0.9992$). ALT concentrations in the range of 0.1 μg/mL to 20 μg/mL exhibit a good linear relationship with peak area. The limit of detection is 0.03 μg/mL, and the limit of quantification is 0.07 μg/mL. The lowest concentration in the calibration curve for this experiment is 0.1 μg/mL, which is greater than the limit of quantification. This meets the analytical requirements and can be used for the determination of ALT concentrations in plasma samples.

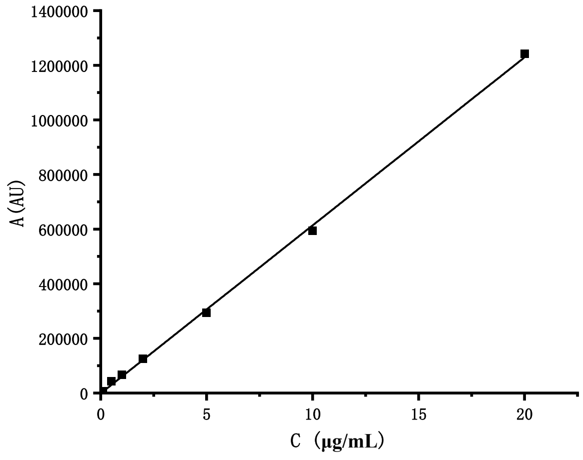

Figure S15 Standard curve for ALT in plasma

S4.5 Precision Determination Experiment

As shown in Table S13, the intra-day and inter-day RSDs for all three concentrations were less than 5%, indicating that this method provides good precision for the determination of ALT within the concentration range of the standard curve and meets the requirements for the assay.

Table S13 Results of intra-day and inter-day precision (n=3)

Actual concentration (μg/mL)	Intraday precision		Interday precision	
	Measured concentration (Mean±SD, μg/mL)	RSD (%)	Measured concentration (Mean±SD, μg/mL)	RSD (%)
0.5	0.49±0.01	2.04	0.48±0.02	4.17
10	9.70±0.10	1.00	9.59±0.18	1.84
20	20.12±0.08	0.41	19.98±0.09	0.45

S4.6 Recovery Rate Determination Experiment

The results of the analysis are shown in Table S14. The sample preparation method described in this chapter yielded high recovery rates; the extraction recovery rates for ALT samples at all concentrations were above 86%, with an RSD of less than 5%, meeting the analytical requirements.

Table S14 Results of the recovery rate (n=3)

Concentration (μg/mL)	Method recovery rate		Extraction recovery rate	
	(Mean±SD)	RSD (%)	(Mean±SD)	RSD (%)
0.5	91.48±2.54	2.78	86.29±3.56	4.12
10	97.84±0.97	1.03	96.74±1.60	1.66
20	100.67±0.29	0.29	98.13±1.70	1.73

S5 Methodology for Investigating Gastrointestinal Absorption Characteristics

S5.1 Establishment of a Method for Determining Phenol Red Content

Weigh 20 mg of phenol red precisely and use K-R to prepare a 400 μg/mL phenol red K-R stock solution. Dilute the stock solution with K-R to prepare a concentration gradient of 4, 6, 8, 10, 16, 20, and 40 μg/mL. Take 0.5 mL of each solution, add 5 mL of 0.2 mol/L NaOH solution, use Kerb's-Ringer's nutrient solution as the blank, and measure the absorbance at 558 nm. Perform a linear regression analysis of absorbance (A) against concentration (C) to plot a standard curve.

The standard curve for phenol red is shown in Figure S16. The regression equation is: A = 0.0165C + 0.0008 (R^2 = 0.9999). The absorbance of phenol red exhibits a good linear relationship with concentration in the range of 4–40 μg/mL.

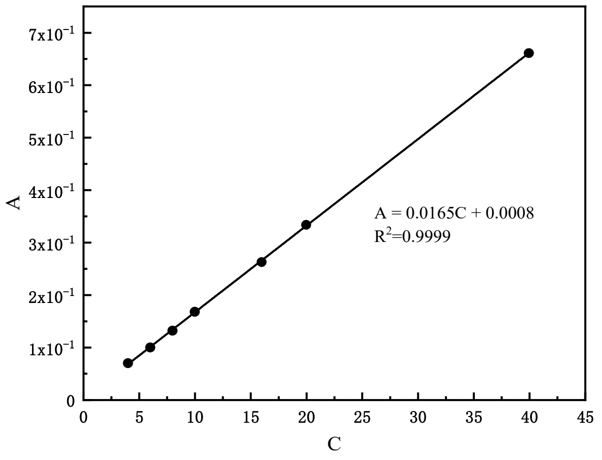

Figure S16 Standard curve of phenol red in phenol red /K-R buffer

S5.1 Establishment of a Method for Determining ALT Concentration in Phenol Red-KR Buffer

S5.1.1 Experiment on Method Specificity

As shown in Figure S17, the ALT peak appears at approximately 4.9 min. The blank phenol red chromatogram shows no signal at the ALT peak position, indicating no interference at the ALT peak position and no interference from endogenous impurities in the vicinity; the method exhibits good selectivity.

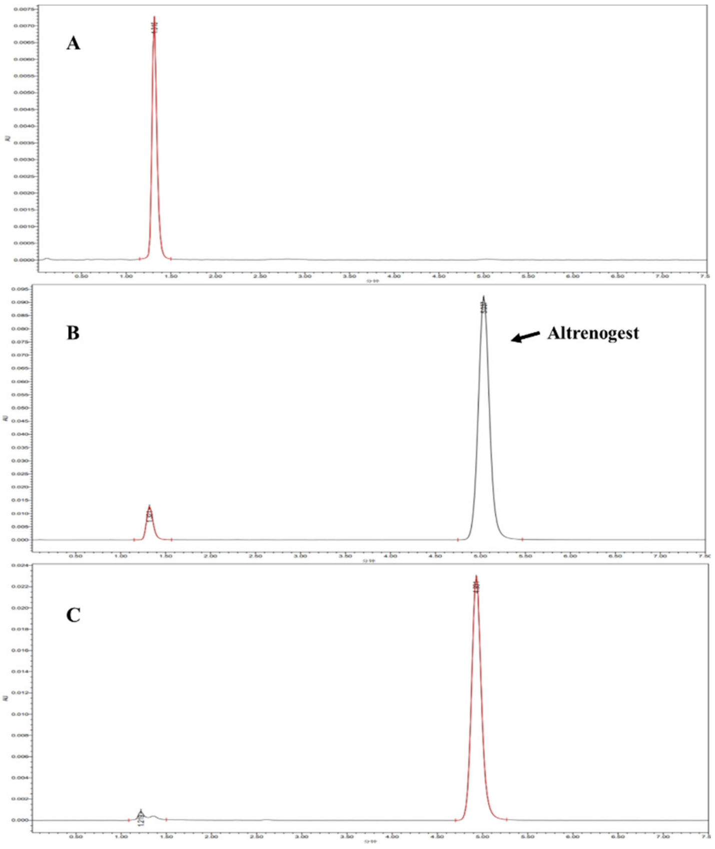

Figure S17 HPLC chromatogram of blank (A) / ALT (B) in phenol red /K-R perfusion and enteral irrigation fluid (C)

S5.1.2 Standard Curve

A series of ALT standard stock solutions with concentration gradients of 1, 2.5, 5, 7.5, 10, 25, 50, 75, 150, and 200 μg/mL were prepared using a blank phenol red K-R reagent solution. These solutions were injected and analyzed in sequence, and a linear regression analysis was performed using peak area (A) against concentration (C).

Based on the regression equation $A = 51{,}521C + 24{,}592$ ($R^2 = 0.9998$), the peak area of ALT shows a good linear relationship with concentration in the range of 1–200 μg/mL.

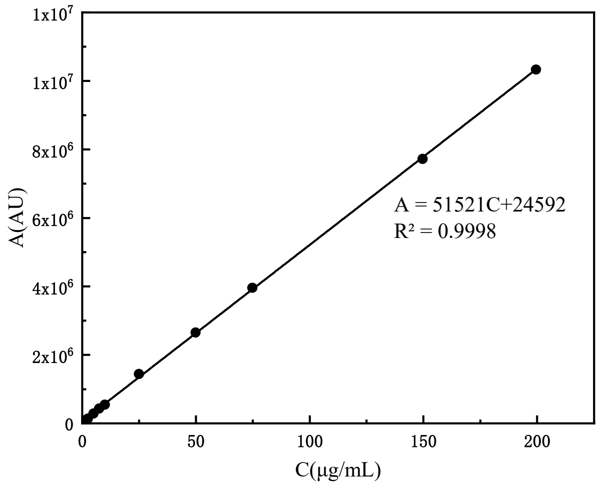

Figure S18 Standard curve of ALT in Phenol Red-KR Buffer

S5.1.3 Precision Determination Experiment

As shown in Table S15, the intra-day and inter-day precision (RSD) for all three concentrations were less than 3%, indicating that this method provides good precision for the determination of ALT within the concentration range of the standard curve and meets the analytical requirements.

Table S15 Results of intraday and interday precision (n=3)

Actual concentration (μg/mL)	Intraday precision		Interday precision	
	measured concentration (Mean±SD, μg/mL)	RSD (%)	measured concentration (Mean±SD, μg/mL)	RSD (%)
10.00	9.67±0.17	1.75	9.35±0.20	2.1
75.00	75.04±1.41	1.88	74.06±1.82	2.46
180.00	179.73±1.05	0.58	180.06±2.58	2.84

S5.1.4 Recovery Rate Determination Experiment

As shown in Table S16, the sample preparation methods described in this chapter yielded high recovery rates, with extraction recoveries all exceeding 90% and RSD values below 5%, meeting the analytical requirements.

Table S16 Results of the recovery rate (n=3)

Concentration (μg/mL)	Method recovery rate		Extraction recovery rate	
	(Mean±SD, %)	RSD (%)	(Mean±SD, %)	RSD (%)
10.00	1.00±0.02	2.02	0.92±0.02	2.17
75.00	1.01±0.01	1.02	0.95±0.02	2.11
180.00	1.01±0.03	2.84	0.91±0.03	3.06

S5.1.5 Testing the Stability of ALT in Perfusion Fluid

The ALT concentration in the perfusion fluid did not change significantly over the 24-hour period and remained relatively stable; furthermore, since this experiment lasted only 2 hours, the effects of various intestinal enzymes on ALT levels can be disregarded.

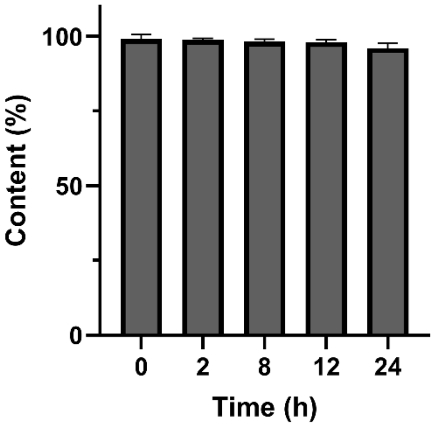

Figure S19 Investigation of ALT stability in internal perfusion fluid in 24 h (n=3)

S6 Acute oral toxicity study of ALT-SNEDDS in rats

S6.1 Animals and housing

Female Sprague-Dawley rats (180 ± 15 g) were used unless otherwise justified. Animals were housed under standard laboratory conditions (temperature 22–25 °C, relative humidity 50–60 %, 12 h light/dark cycle) with free access to food and water, and were acclimatised for at least 7 days before the experiment. All animal procedures were reviewed and approved by the Ethical Scientific Committee of Sichuan Agricultural University (Chengdu, China; Approval No. SAU-20250036).

S6.2 Study design and dosing

Rats were randomly divided into four groups (n = 6 per group): (i) normal saline control; (ii) blank SNEDDS control (excipient control); (iii) ALT-oil solution; and (iv) ALT-SNEDDS. After an overnight fast with free access to water, each formulation was administered once by oral gavage at a dose equivalent to 40 mg ALT kg^{-1}; the control group received an equal volume of normal saline. The blank SNEDDS control received the same amount of the drug-free pre-concentrate. Food was returned 4 h after dosing.

S6.3 Clinical observation and body weight

Animals were observed continuously during the first 4 h after dosing and then at least twice daily for 7 days. The observations covered changes in fur, skin, eyes, mucous membranes, respiration, salivation, lacrimation, piloerection, tremor, diarrhoea, lethargy, posture and mortality. Body weight was recorded on days 0, 1, 3 and 7.

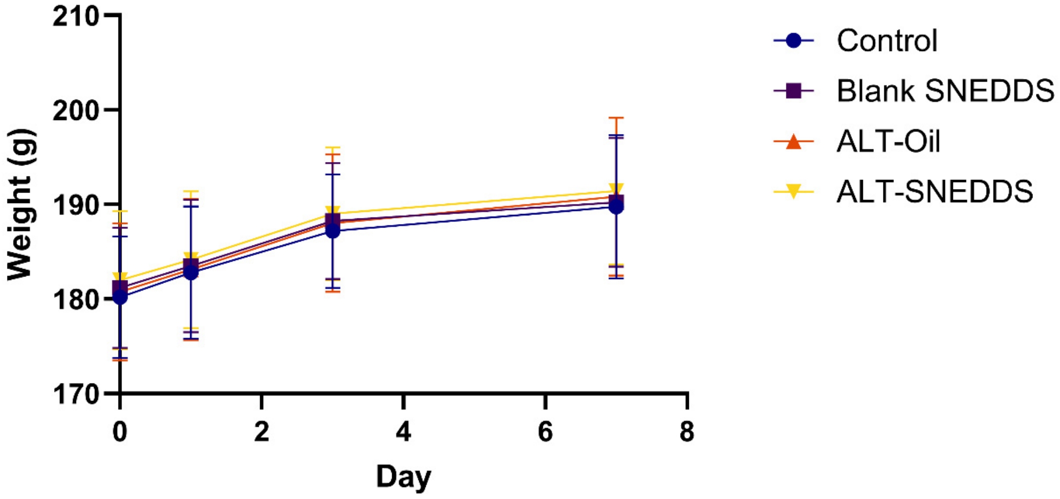

Figure S20 Body weight (g) of rats in each group during the 7-day observation period (mean ± SD)

S6.4 Gross necropsy and organ coefficients

On day 7, all surviving rats were deeply anesthetized by intraperitoneal injection of tribromoethanol (Avertin; 1.25% w/v, 400 mg/kg). The depth of anesthesia was confirmed by loss of the righting reflex, absence of a withdrawal response to toe pinch, and absence of the corneal reflex. After deep anesthesia was verified, rats were euthanized by cervical dislocation performed by trained personnel. Death was confirmed by the cessation of spontaneous respiration and heartbeat for at least 5 min, together with pupil dilation. The heart, liver, spleen, lung, kidneys, stomach and intestine were excised, examined macroscopically for lesions, washed with ice-cold saline, blotted dry and weighed. Organ coefficients were calculated as (organ weight / body weight) × 100 %.

Table S17 Organ coefficients (%) of rats (mean ± SD)

Groups	Organ coefficients (%)(mean ± SD)				
	Heart	Liver	Lung	Spleen	Kidneys
Control	0.38±0.03	5.09±0.38	0.57±0.05	0.29±0.05	0.85±0.04
Blank SNEDDS	0.38±0.02	5.06±0.34	0.57±0.04	0.28±0.02	0.86±0.06
ALT-Oil	0.39±0.04	5.14±0.25	0.61±0.04	0.27±0.04	0.83±0.04
ALT-SNEDDS	0.37±0.04	5.09±0.20	0.57±0.03	0.29±0.03	0.86±0.03

S6.5 Histopathological examination

The excised organs were fixed in 10 % neutral buffered formalin for at least 24 h, dehydrated in a graded ethanol series, cleared in xylene, embedded in paraffin, sectioned at 4–5 μm, and stained with haematoxylin and eosin (H&E). Sections were examined under a light microscope and photographed at 20×.

Seven days after a single oral administration, the heart, liver, lung, spleen and kidney were collected and examined on haematoxylin and eosin (H&E)-stained sections. In the control group, all examined organs displayed normal tissue architecture. Compared with the control group, no apparent histopathological abnormalities were observed in the ALT-SNEDDS group. Specifically, the myocardial fibres were regularly arranged without necrosis, degeneration or inflammatory infiltration; the hepatic lobules and cords were intact, with no fatty degeneration, focal necrosis or fibrosis; the alveolar walls were thin and clear, without exudate or interstitial inflammation; the splenic red and white pulp were well demarcated with normal lymphoid follicles; and the renal glomeruli and tubules were morphologically normal, with no interstitial inflammation or fibrosis. No necrosis, inflammatory cell infiltration, fatty degeneration, fibrosis or haemorrhage was found in any of the examined organs.

These results indicate that a single oral administration of ALT-SNEDDS at 40 mg/kg, did not induce detectable acute toxicity or histopathological changes in the major organs examined, supporting the acceptable acute tolerability of the formulation.

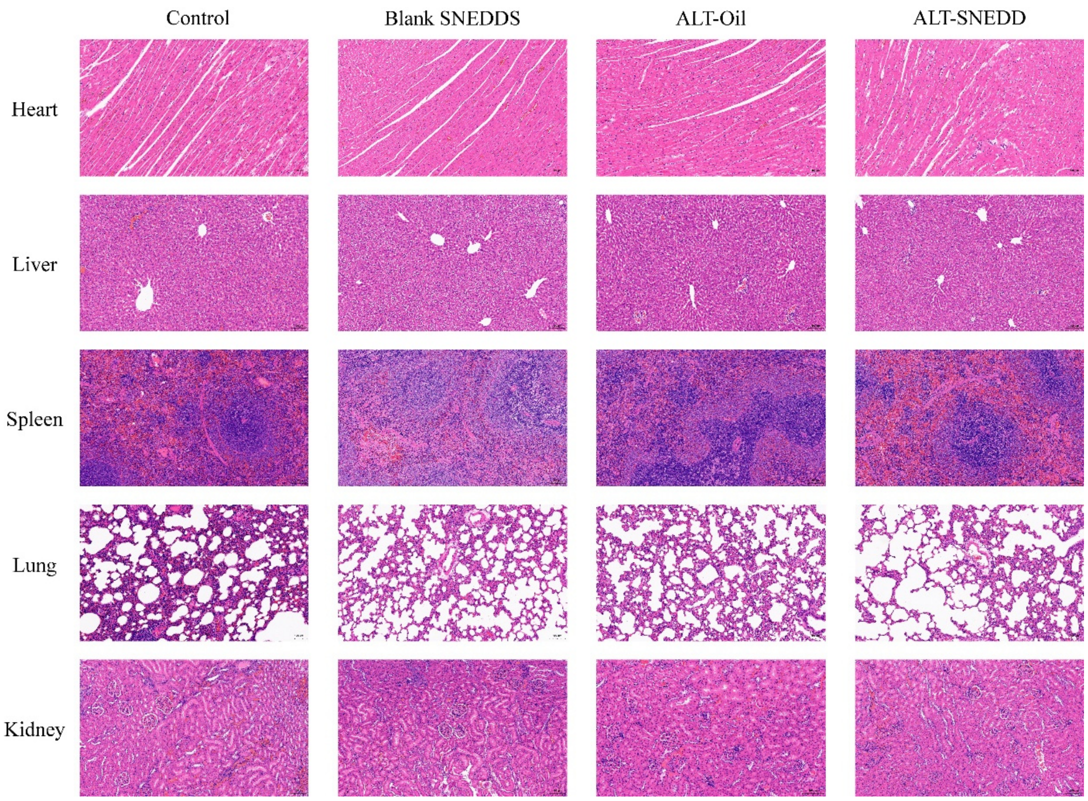

Figure S21 Representative H&E-stained sections of the heart, liver, spleen, lung and kidney from each group
